# Chemical, Nutritional, and Antihyperglycemic Studies on Sonora Gum

**DOI:** 10.1021/acsomega.5c02073

**Published:** 2025-06-18

**Authors:** Araceli Pérez-Vásquez, Vanya Meneses-Pérez, Valeria Reyes-Pérez, Laura Flores-Bocanegra, Manuel Rangel-Grimaldo, Edelmira Linares, Robert Bye, Rachel Mata

**Affiliations:** † Facultad de Química, 7180 Universidad Nacional Autónoma de México, Ciudad de México 04510, Mexico; ‡ Instituto de Química, Universidad Nacional Autónoma de México, Ciudad de México 04510, Mexico; § Instituto de Biología, 7180 Universidad Nacional Autónoma de México, Ciudad de México 04510, Mexico

## Abstract

The Rarámuri Indigenous people of Chihuahua, Mexico, use the gum of *Tachardiella fulgens* (“arí” or Sonora gum) as a medicinal agent and food. Thus, this work established the chemical composition, nutritional value, potential toxicity, and antihyperglycemic action of Sonora gum. Using spectroscopic, spectrometric, and chromatographic methods including UHPLC-MS analysis, it was demonstrated that arí contains anthraquinones (laccaic acids A, B, and E, xantholaccaic acid B, emodin, and erythrolaccin), farnesol, and nerolidol derivatives including (2*Z*,6*E*,10*E*)-farnesol-12-oic acid (**1**), a new natural product, crocinervolide, and (6*E,*10*E,*3*S*)-nerolidol-12-oic acid. The volatilome, determined by headspace solid-phase microextraction coupled with GC-MS analysis, was characterized by lilac derivatives, fatty acids, and cedrene alcohols. Using official AOAC and the US Department of Agriculture methods, the proximal analysis revealed that arí contains high dietary fiber and vitamins B2 and B3. In addition, an aqueous extract of arí significantly reduced the postprandial peak at the highest dose tested (316 mg/kg) in healthy and hyperglycemic mice during an oral glucose tolerance test, meaning that arí increases glucose utilization in the hyperglycemic condition. According to the Lorke method, the LD_50_ of the extract was estimated to be above 5 g/kg, indicating that the extract is practically nontoxic. This work represents the first comprehensive study of the chemical, pharmacological, and nutritional aspects of arí, a valuable Mexican food. The findings reveal that arí is a source of bioactive specialized metabolites, including anthraquinones and sesquiterpenoid acids, lilac aldehydes, fatty acids, and micro- and macronutrients. Thus, the use of arí as both food and medicine has a rational basis and enhances knowledge of Mexican ancestral foods and medicine.

## Graphical abstract

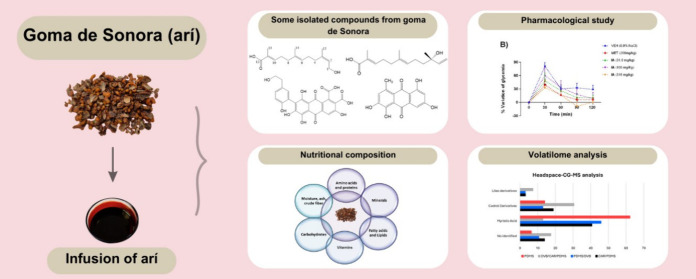

## 1 Introduction

The Sonoran gum is a resinous material produced by the North American lac scale insect *Tachardiella fulgens* Cockerell (Hemiptera: Kerriidae)^1^ that lives on the twigs of *Coursetia glandulosa* A. Gray, a small tree in the Fabaceae family, commonly called rosary babybonnets, samó or sweet stick (Figure 1). Even though the host tree grows from southern Arizona, along the Pacific slope of Mexico, to northern Central America, the lac produced by the immobile female with the mutualistic aid of ants (*Myrmecocystus* sp.; Hymenoptera: Formicidae) is known only in northwestern Mexico and adjacent parts of the USA.^2^

**Figure 1:**
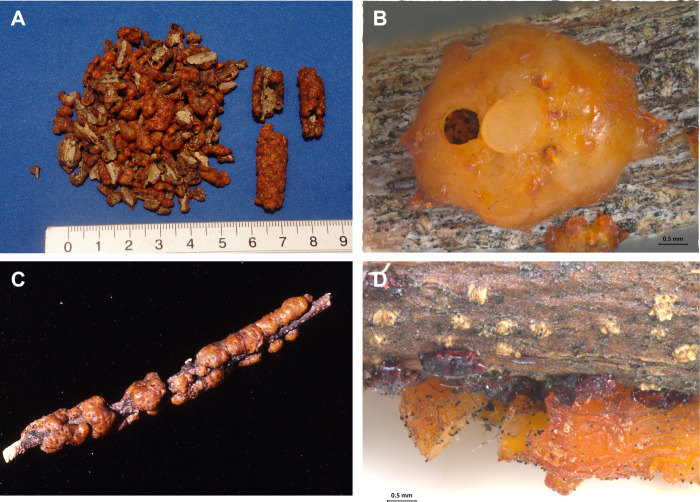
(A) Shelled form (which is the presentation of acquisition) and wrapped form of *T. fulgens*; (B) Adult female with lac test (resin cover) from which the nymphs emerged; (C) insect exudates around *C. glandulosa* stem; (D) magnified view of twig of *C. glandulosa* with the exoskeleton of insect *T. fulgens*. Photos were by Robert Bye.

According to Cockerell^1^ in the first scientific description, the lac consists of the female scales of *T. fulgens*, massed together to form an irregular, bright, reddish-orange coating approximately 4 mm thick, surrounding the twig (Figure 1). The Mexicans used the lac for traditional medicinal purposes related to stomach issues, referring to it as “gomea”, and for repairing pottery. Cockerell also noted that Mexicans made a clear distinction between *T. fulgens* and *T. larreae* Comstock, which lacked medicinal properties. A single scale of *T. fulgens* measures about 5 mm long and 4 mm wide, featuring a noticeable curved and shiny dorsal bump. The exudate is hard and encases the insect bodies and eggs.^1,3^

During the 18th century, the “gomilla colorada” from Sonora was used throughout New Spain as an antidote against poisonous wounds and animal bites.^4^ The importance of this biocultural resource changed over time.^5^ Anthropological studies in Chihuahua during the late 19th and early 20th centuries documented the use of arí as food among the Tarahumara. Carl Lumholtz reported that arí’s “taste is sweetish acid, not particularly pleasant to the palate, but very refreshing in effect”.^5^ Later, Robert Zingg reported: “They [Tarahumara] are inordinately fond of it as a relish ··· I myself have eaten it, and it is no stranger than bird’s-nests soup, which I used to get in the Philippines”.^6^

In contemporary Mexico, the Tarahumara (Rarámuri) is the largest of the four surviving Indigenous peoples (“Pueblos Indígenas”) in the Northern Sierra Madre Occidental of Mexico. Linguistically, they belong to the subfamily Cahíta-Ópata-Tarahumara of the Uto-Aztecan language family. Their transhumance character allows the utilization of biocultural resources along the temperate-tropical ecological gradient.^7–12^ From the host tree samó growing in the tropical canyons, the Rarámuri collect the arí for domestic uses as medicinal agent, food, sealant for pottery, and adhesive as well as a trade item in the local shops of the mountains and the urban markets in Chihuahua, Sonora and Sinaloa under the name of “goma de Sonora” ^7–10^ It is a popular ingredient in aguachile in combination with lime juice, chiles, and cilantro that is consumed as a spicy, acidic cold soup or as a sauce used to marinate seafood and fish. As a medicinal agent, the decoction of arí is recommended for treating colds, headaches, diarrhea, dysentery, stomach aches, diabetes, and hangovers caused by excessive drinking.^7–12^

Previous chemical investigations using LC-MS of the stick lac of *T. fulgens* and *T. larreae* obtained from the Smithsonian National Museum of Natural History at Beltsville/Systematic Entomology Laboratory, Department of Agriculture, Agricultural Research Service (USDA ARC) indicated the presence of laccaic acid B and another similar compound with one less hydroxy group.^8^ Later on, a GC-MS study of lac branches with *T. fulgens* and *T. larreae* detected the presence of 44 and 23 compounds, respectively, which were not unequivocally identified.^9^ The authors concluded that the samples were chemically complex and contained long-chain fatty acid esters. Other constituents reported in the same samples were nerol methyl ether, *cis*-*Z*-α-bisabolene epoxide, a few hydrocarbons, and some non-natural products such as the herbicide tridemorph. In both studies, the chemical characterization was not rigorous because no retention indexes or retention times, spectrometric analysis, or exact mass values were provided.^9^ Finally, the same group used Fourier transform infrared spectroscopy, UV-induced visible fluorescence, and microchemical testing studies to detect the lac of *T. fulgens* and *T. larreae* in a few archeological artifacts.^9,10^ Hence, the chemistry and biological properties of lac from *T. fulgens* remain an open question.

It is worth mentioning that the Oriental insect *Kerria lacca* Kerr, which belongs to the same insect family as *T. fulgens* and *T. larreae*, produces lac, a raw material used in India for shellac manufacturing. Shellac is widely used in food coatings, cosmetology, and pharmaceutical delivery systems; the lac produced by *K. lacca* has been extensively studied from a chemical perspective.^11,13^

Considering the introductory information, this work aims to establish the chemical composition, nutritional value, potential toxicity, and antihyperglycemic properties of Sonora gum, promoting its rational use and conservation, while contributing to the knowledge of Mexican ancestral foods and medicines. To date, there has been no comprehensive study of this valuable food from Northern Mexico. The pharmacological testing was selected based on the high prevalence of diabetes in Mexico, and the unverified use of natural sources, including arí, for the treatment of diabetes.^14^

## 2 Materials and Methods

### 2.1 Chemicals and Reagents

UHPLC grade acetonitrile, water, and analytical reagent (AR) grade solvents for extraction (acetone, methanol, hexane, ethyl acetate, chloroform, and dichloromethane) were purchased from J.T. Baker (Avantor, Radnor, PA, USA). The homologous series of *n*-alkanes used as standards in GC-MS analysis consisted of a mixture of C_7_–C_30_ saturated alkanes (1000 μg/mL for each component in hexane), provided by Supelco (Sigma-Aldrich Quimica, Toluca, Mexico). Carotenoids (β-carotene and lutein), a FAME mixture (C_8_–C_24_), octadecane, and streptozotocin were obtained from Merck (Sigma-Aldrich Quimica, Toluca, Mexico).

### 2.2 General Experimental Procedures

Optical rotations were measured by using a PerkinElmer 343 polarimeter at 22 °C. IR spectra were recorded on a PerkinElmer Spectrum 400 FTIR/FIR spectrophotometer. 1D and 2D NMR spectra were recorded on a Varian VNMRS instrument operating at a radio frequency of 400 and 100 MHz, respectively, and analyzed using MNova software (version 12.0.2). Residual D_2_O, CH_3_OH-*d*_4_, or DMSO-*d*_6_ solvent signals were used as reference. High-resolution mass spectrometry (ESI-TOF) was conducted using an Agilent Technologies 6530 Accurate-Mass Q-TOF LC/MS equipment.

Thin-layer chromatography (TLC) was performed using Merck Silica Gel GF_254_, 0.25 mm thickness, and multiple elution systems, including EtOAc-MeOH-H_2_O (8.5:1:0.5), CH_2_Cl_2_-MeOH (9:1 or 8.5:1.5), CHCl_3_-MeOH (8.5:1.5; with three drops of AcOH), and *n*-butanol-2-propanol-MeOH-H_2_O (1.5:6:1:1.5). Preparative TLC analyses were developed on precoated glass sheets (silica gel G F_254_, 0.5 mm) using CH_2_Cl_2_-MeOH (9:1) as the elution system. Column chromatography was performed using silica gel 60 (Merck), Sephadex LH-20 (Merck), or Diaion HP-20 (Merck), and the latter two were previously conditioned with methanol.

### 2.3 Sonora Gum and Plant Material

Robert Bye and Edelmira Linares taxonomically identified and attained all materials used in this work, in two different regions of Chihuahua, Mexico. The first batch (I) of Sonora gum was obtained from Barranca Urique (municipio Urique) in September 2016, and the second batch (II) came from Barranca Sinforosa (municipio Guachochi) in February 2017. Batch II (100 mg) was used for nutritional analysis. Voucher specimens were deposited in the National Herbarium of México (MEXU) as Bye 38403 & Linares and Bye 38566, respectively. The stem bark of *Coursetia glandulosa* was collected in Barranca Batopilas, Chihuahua, in November 2020. The voucher specimen was deposited in MEXU as Bye, Linares & Nevarez 39947.

### 2.4 Extraction and Isolation of Compounds **1**–**6** from Sonora Gum

Two and a half grams of fine powder (ground in a mortar) were extracted with 150 mL of boiling water and distilled for 30 min. After filtration, the extract was concentrated under a vacuum to yield 0.15 g. This process was repeated to yield 5.0 g of aqueous extract (6.1% w/w, IA).

IA (4.0 g) was dissolved in 100 mL of H_2_O and separated by reversed-phase chromatography on a Diaion HP-20. The column was eluted using different percentages of MeOH–H_2_O_dd_ to obtain five fractions (F1, 0%; F2, 75:25; F3, 50:50; F4, 25:75; and F5,100%) of 300 mL, which were concentrated in vacuo. The fraction F5 (102.5 mg) was subjected to further chromatography on Sephadex LH-20, eluted with a gradient of increasing polarity of MeOH–H_2_O to afford 115 fractions that were pooled into 11 secondary fractions (F5.1–F5.11) according to their chromatographic profiles observed in the TLC. From fraction F5.8, 1 mg of **5** was obtained, and from fraction F5.9, 2.8 mg of **4**. Preparative TLC on silica gel of F5.3 (62.2 mg), eluted with CH_2_Cl_2_-MeOH (9:1), yielded 8 mg of **3**, 6.5 mg of **2,** and 6.0 mg of **1**. Fraction F2 was analyzed by UHPLC-MS/UV, which detected and characterized the compounds listed in Table 2. In addition, F2 (80 mg) was fractionated with a Sephadex LH-20 column [80 mL; H_2_O-acetone (2:8)] to yield 8 mg of compound **6.**

#### 2.4.1 (2*Z*,6*E*,10*E*)-Farnesol-12-oic Acid (**1**)

Colorless, viscous oil; UV (MeOH) λ_max_ (log ε) 220 (2.30) nm; IR (FT-IR-ATR) ν_max_ 3346, 2924, 1687, 1416, 1276, 993 cm^–1^; For ^1^H NMR (CH_3_OH-*d*_4_, 400 MHz) and ^13^C NMR (CH_3_OH-*d*_4_, 100 MHz) data, see Table 1; HRMS (ESI-TOF) *m*/*z* 275.1604 [M + Na]^+^ (calcd for [C_15_H_24_O_3_Na]^+^, 275.1617), *m*/*z* 235.1689 [M – OH]^+^ (calcd for [C_15_H_23_O_2_]^+^, 235.1692).

**Table 1:** **^1^** H and **^13^** C NMR Spectroscopic Data of Compounds **1** and **2** (δ in ppm, *J* in Hz)

	**1** ^a^		**2** ^a^	
	δ_C_	δ_H_	δ_C_	δ_H_
1	59.2	4.10 (dd, 1.2, 6.9, 2H)	112.0	a: 5.21 (dd, 1.6, 17.4, 1H) b: 5.04 (dd, 1.6, 10.8, 1H)
2	125.8	5.4 (tq, 1.5, 6.8, 1H)	146.3	5.93 (dd, 10.8, 17.4, 1H)
3	139.6	−	73.8	−
4	32.9	2.14–2.18 (m, 2H)	48.3	1.53 (ddd, 10.2, 6.7, 2.0, 2H)
5	27.6	2.14–2.18 (m, 2H)	23.7	2.05 (m, 2H)
6	125.8	5.21–5.24 (m, 1H)	126.6	5.18 (m, 1H)
7	135.6	−	135.0	−
8	39.3	2.18–2.14 (m, 2H)	39.3	2.13 (t, 7.3, 2H)
9	28.2	2.35 (q, 7.4, 2H)	28.2	2.32 (ddd, 1.0, 7.1, 8.0, 2H)
10	143.0	6.77 (tq, 1.5, 7.3, 1H)	143.5	6.77 (tq, 1.4, 7.4, 1H)
11	129.3^b^	−	129.0	−
12	172.1^b^	−	171.8	−
13	12.5	1.85 (bs, 3H)	12.5	1.82 (bs, 3H)
14	15.9	1.69 (bs, 3H)	15.9	1.65 (bs, 3H)
15	23.7	1.78 (bs, 3H)	27.6	1.26 (bs, 3H)

a Recorded at 400 MHz, 100 MHz in MeOH-*d*_4_.

b Detected by HMBC.

#### 2.4.2 (6*E*,10*E*,3*S*)-Nerolidol-12-oic Acid (**2**)

Colorless, viscous oil; [α]^25^_D_ + 8.0 (*c* 0.15, MeOH); UV (MeOH) λ_max_ (log ε) 220 (2.70) nm; IR (FT-IR-ATR) ν_max_ 3374, 292 7, 1685, 1413, 1279, 919 cm^–1^; For ^1^H NMR (CH_3_OH-*d*_4_, 400 MHz) and ^13^C NMR (CH_3_OH-*d*_4_, 100 MHz) data, see Table 1; HRMS (ESI-TOF) *m*/*z* 275.1607 [M + Na]^+^ (calcd for [C_15_H_24_O_3_Na]^+^, 275.1617), *m*/*z* 235.1683 [M – OH]^+^ (calcd for [C_15_H_23_O_2_]^+^, 235.1692).

### 2.5 Extraction and Isolation of Compounds **12**–**15** from the Stem Bark of *C. glandulosa*

A dried and ground plant material (1.5 kg) was macerated with 8 L of acetone for 21 days. The resulting extract (AE) was evaporated to dryness (18.0 g) and subjected to an open-column on silica gel (deactivated with 10% H_2_O_dd_) with a gradient of hexane-EtOAc and EtOAc-MeOH (100:0 → 0:100 → 80:20) to afford 82 fractions, which were pooled into 15 primary fractions (C1–C15), according to their chromatographic profiles observed in the TLC. From the primary fraction C15, 548.6 mg of (+)-pinitol (**12**) were isolated. C1 yielded 129.8 mg of stigmasterol (**13**); fraction C12 generated 45.6 mg of β-d-glucositosterol (**14**); and fraction C6 (130 mg) yielded upon crystallization from hexane 4.0 mg of ursolic acid (**15**). The same procedure as that for IA was used to prepare the aqueous extract of the stem of *C. glandulosa*. Thus, 2.5 g of ground plant material was extracted with 150 mL of boiling water and distilled for 30 min. After filtration, the extract was concentrated in vacuo to obtain 205 mg of a dry extract (8.2% w/w dry weight).

### 2.6 HS-SPME Extraction of Sonora Gum

Three hundred milligrams of the ground and dried insect lac exuded, along with sodium chloride (300 mg), and HPLC grade water (15 mL) was transferred into a 40 mL clear glass headspace vial (Supelco, Sigma-Aldrich) hermetically sealed with a polypropylene hole-cap and PTFE/coated silicone septa. Four fibers (polydimethylsiloxane (PDMS), divinylbenzene/carboxen/polydimethylsiloxane (DVB/Car/PDMS), polydimethylsiloxane/divinylbenzene (PDMS/DVB), and carboxen/polydimethylsiloxane (Car/PDMS) were used. For the analysis, the experimental conditions were set: extraction temperature 60 °C, extraction time 15 min, and stirring rate 450 rpm. After sampling with the headspace method (HS), the SPME fibers were directly inserted into the CG injector port and thermally desorbed for 14 min at 300 °C. All samples were analyzed in triplicate.

### 2.7 GC-MS Analysis

A PerkinElmer Clarus 680 SQ8C GC/MS was employed for gas chromatography–mass spectrometry analysis, and an Agilent Tech HP-5MS column (30 m × 0.25 mm × 0.25 μm) was used to separate analytes. Helium (99.999%) was used as a carrier gas at a 1 mL/min flow rate. The column oven temperature was initially set at 40 °C and was held for 3 min, subsequently ramped at 20 °C/min to 300 °C, and held for 9 min. Splitless injection was used for the analysis. The mass spectrometer was operated with the transfer line set at 250 °C, ion source at 200 °C, and quadrupole at 150 °C. Electron impact ionization was employed, with electron energy at 70 eV, and a mass range set at 33–600 *m*/*z* in full-scan acquisition mode. The relative intensity percentage (%) of each component in GC was calculated using their peak areas in relation to the total area of the detected peaks.

Volatile components were characterized by comparing their retention indices and mass spectra with those of the NIST library database (NIST Chemistry WebBook).^15,16^ A mixture of *n*-alkanes (C_7_–C_30_) was injected under conditions identical to those of the sample, and the van Den Dool and Kratz method was applied to calculate the retention indices. Octadecane (C_18_H_38_) was used as the internal standard to correct the retention indices.

### 2.8 Ultrahigh-Performance Liquid Chromatography Coupled to Mass Spectrometry (UHPLC-MS)

UHPLC-MS analyses were performed using a Waters Acquity UPLC system equipped with a quaternary pump, an autosampler, PDA, ELS and SQD2 detectors, and an electrospray ionization energy source. A UPLC BEH C_18_ column (1.7 μm; 50 × 2.1 mm) was used. The column temperature was maintained at 40 °C. The column was eluted with a mobile phase of CH_3_CN-H_2_O_dd_, both added with 0.1% formic acid and with the following gradient program: 0 min 15% CH_3_CN to 8 min 100% CH_3_CN to 10 min 15% CH_3_CN. The flow rate was set to 0.3 mL/min. UV/vis spectra were recorded in the 192–500 nm range. A Thermo LTQ Orbitrap XL mass spectrometer (ThermoFisher, San Jose, CA, USA) equipped with an electrospray ionization source was used. Source conditions in positive-ionization mode were set at 275 °C for the capillary temperature, 4.5 kV for the source voltage, 20 V for the capillary voltage, and 95 V for the tube lens. For the negative ionization mode, the source conditions for temperature were 3.5 kV for the source voltage, 42 V for the capillary voltage, and 110 V for the tube lens. In both modes, two scan events were carried out: full-scan (100–2000) and ion-trap MS/MS of the most intense ion from the parent mass list utilizing CID with a normalized collision energy of 30. External instrument calibration was performed using an LTQ ESI positive-ion calibration solution containing caffeine at a concentration of 20 μg/mL. For the ESI negative-ion calibration, sodium dodecyl sulfate (2.9 μg/mL) was added to the LTQ ESI calibration solution instead of caffeine. The full scan chromatograms and spectra were acquired over the *m*/*z* range of 100–1500. The data was analyzed using MNova software (v12.0.2, Mestrelab Research S.L.). The exact mass (considering Δ_ppm_ values <5 ppm) and the maximum wavelengths observed were used as characterization criteria. Thus, the identification of the compounds was conducted by comparing experimental data with theoretical data reported by databases such as CAS SciFinder and the Dictionary of Natural Products (ChemNetBase). Laccaic acid B, isolated from the arí, was the only compound that could be unequivocally identified by coelution.

### 2.9 Food Analysis of Sonora Gum

The dry and pulverized material (100 g, bath II) was analyzed to determine the contents of vitamins, minerals, lipids, fatty acids, and polyols in the sample. In addition, a proximal analysis of the content was realized. These analyses were conducted at the Unidad de Servicios para la Industria de Alimentos (USIA) at Facultad de Química, UNAM.

In the proximal analyses, moisture (930.04), protein (978.04), fat (ethereal extract; 920.85), ash (930.05), and total dietary fiber (993.21) were analyzed following AOAC Official Methods.^17^ Carbohydrate content was quantified following the protocol described in the US Department of Agriculture, Agricultural Research Service, Nutrient Data Laboratory.^18^ The conversion factors mentioned in the FAO Food and Nutrition Paper 77 were used to calculate the energy intake.^19^ The minerals Ca, Mg, Na, Cu, Fe, Zn, and K were determined using AOAC official method 991.25, and in the case of P, method 931.01 was used.^17^ The determination of vitamins A, D, and E was carried out according to the method described by Barnett, vitamin C by Zapata and Dufour, and complex-B vitamins by Albadlá-Hurtado et al.^20–22^ The determination of amino acids was carried out according to the methodology described by Perucho et al.^23^ The polyol content (xylitol, sorbitol, and pinitol) was determined by high-performance liquid chromatography coupled to a charged aerosol detector described by Grembecka et al.^24^ Finally, the fatty acids were determined by gas chromatography using the Official method AOAC 996.06.^17^

### 2.10 Oral Glucose Tolerance Test (OGTT)

The experimental procedures involving animals comply with the guidelines outlined in the Mexican Official Standard for the Care and Use of Animals (NOM-062-ZOO-1999) and the International Ethical Guidelines for the Care and Use of Laboratory Animals. The protocols for the use of animals in the OGTT were approved by the Ethical Committee of the Facultad de Química, UNAM (FQ/CICUAL/546/24). Six-week-old male CD-1 mice (25–40 g, body weight) were used for pharmacological testing. Animals were purchased from Crculo ADN, SA de CV. The animals were maintained at a constant temperature (25 °C) with a 12 h light/dark cycle and provided unrestricted access to water and food pellets (Lab Diet 5001 Rodent Diet). Blood samples were collected through a small incision at the tip of the mouse’s tail, and glucose levels were measured using a One Touch Select Plus Flex glucometer (Life Sacan, Gubelstrasse, Switzerland). The induction of hyperglycemia in mice was carried out according to Rebollar-Ramos and co-workers,^25^ where mice were administered by intraperitoneal injection with three consecutive doses of streptozotocin (40 mg/kg) in buffer solution (citrates 100 mM, pH = 4.5). After 21 days, mice with blood glucose levels exceeding 200 mg/dL were classified as hyperglycemic and included in the study. All experiments were done with fasted mice 4 h before the experimental handling. After the experiments, the mice were euthanized by hypoxia in a CO_2_ chamber. The experiment was conducted in normoglycemic and hyperglycemic mice; in both cases, the protocol was executed as follows. Mice were separated into five groups (*n* = 6), and all were administered *p.o*. Before administration of the treatments, basal glucose levels were measured. Group I received the vehicle solution (VEH, saline solution with 10% Tween 80); group II, with the positive control metformin (MET, 200 mg/kg); and group III–V, with IA at three different doses: 31.6, 100.0, and 316.0 mg/kg. Thirty minutes after administering the treatment, an oral glucose (1 g/kg) load was given to all groups. Blood glucose levels were determined 30, 60, 90, and 120 min after the glucose load. The percentage of glycemic variation (%) was calculated relative to the basal level as follows:

$$\%\mathrm{variation}\;\mathrm{of}\;\mathrm{glycemia}=\frac{G_{t}-G_{i}}{G_{i}}\times 100\%$$

Results are expressed as the mean ± standard error (SEM) of glycemia variation percentage. Statistical significance (*p < 0.05, **p < 0.01, ***p < 0.001, and ****p < 0.0001) was assessed with the GraphPad Prism software (version 8.0.2) using one-way or two-way ANOVA test followed by Dunnett’s post hoc test.

### 2.11 Acute Oral Toxicity in Mice

The acute toxicity of the aqueous extract of Sonora gum (IA) was evaluated in mice (see above) following the Lorke protocol.^26^ This assay was also approved by the local ethics committee (FQ/CICUAL/549/24). In two independent phases, IA was dissolved in a saline solution and administered by an intragastric route. In each case, the mice were divided into four groups (*n* = 3). During the initial phase, the animals received doses of 10, 100, and 1000 mg/kg. In the second phase, doses of 1600, 2900, and 5000 mg/kg were administered. Control animals were administered with saline solution with 10% Tween 80. The animals were weighed daily over 14 days at each stage. At the end of the experiment, the animals were euthanized to collect the lungs, heart, kidneys, and liver for the assessment of macroscopic organ damage. The mice were observed to identify acute toxic effects, changes in behavioral patterns, or mortality.

## 3 Results and Discussion

### 3.1 Isolation of Compounds **1**–**6** from Sonora Gum

First, we analyzed the aqueous extract obtained by infusion (IA) to conduct chemical analysis. This preparation was selected considering its use in traditional aqueous beverages (tesgüino and “aguachile”). The extract was fractionated using chromatographic procedures to yield compounds **1**–**6** (Figure 2), which included one farnesol derivative, two nerolidol derivatives, and three anthraquinones.

**Figure 2:**
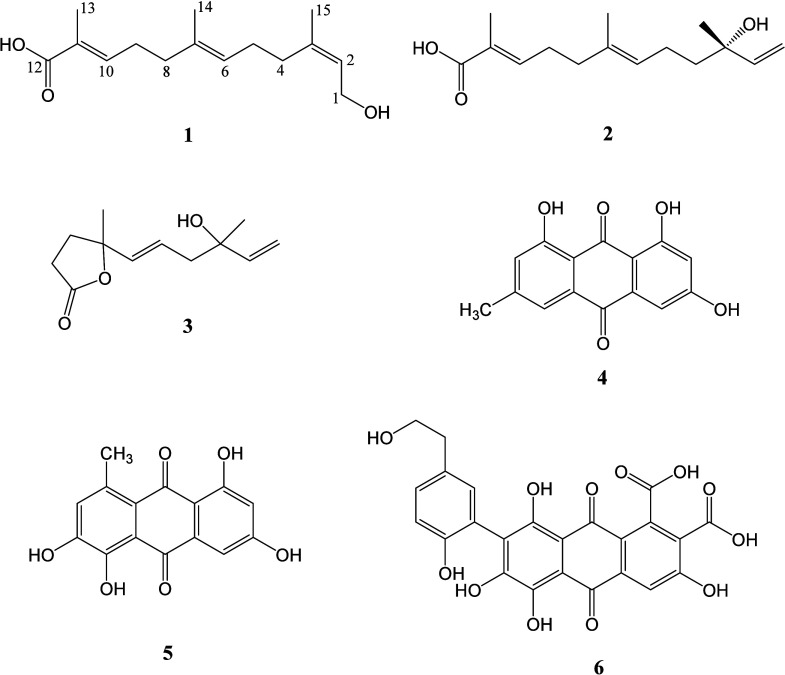
Structures of **1**–**6** isolated from the Sonora gum.

Compound **1** is a new farnesol derivative, and a detailed spectroscopic analysis established its identity. It was obtained as a viscous colorless oil. HRESIMS established its molecular formula as C_15_H_24_O_3_, with an unsaturation index of 4. The IR spectrum showed characteristic bands for conjugated acid (1685 cm^–1^) and carbinolic (3374 cm^–1^) functionalities. The NMR spectra were similar to those of compound **2**, differing mainly in the resonances for C-1, C-3, and C-15 nuclei (Table 1). Thus, the signals of the typical terminal double bond of *E*-nerolidol as seen in **2** [δ_Η_/δ_C_ 5.21 (dd, *J* = 1.6, 17.4 Hz, H-1a), 5.04 (dd, *J* = 1.6, 10.8 Hz, H-1b)/ 112.0 (C-1); 5.93 (dd, *J* = 10.8, 17.4, H-2)/ 146.3 (C-2); δ_C_ 73.8 (C-3)] were replaced by those of terminal allylic alcohol [δ_Η_/δ_C_ 4.10 (dd, *J* = 1.2, 6.9, Hz, H-1)/ 59.2 (C-1); 5.4 (tq, *J* = 1.5, 6.8 Hz, H-2)/ 125.8 (C-2) and 139.6 (C-3)]. The remaining signals, except for C-4, were almost identical and were assigned using the COSY, HSQC, HMBC, and NOESY experiments (Figure 3). Therefore, compound **1** was characterized as (2*Z*,6*E*,10*E*)-farnesol-12-oic acid (**1**).

**Figure 3:**
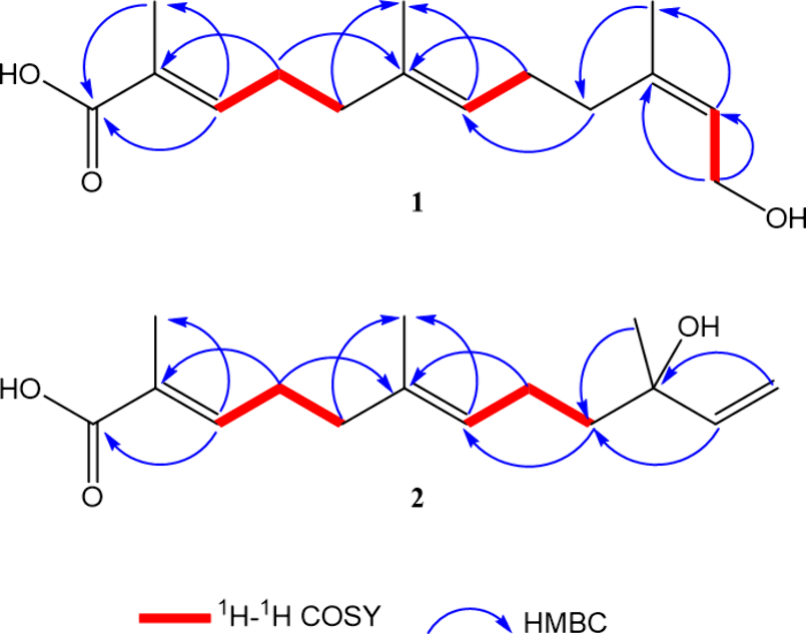
Key ^1^H–^1^H COSY and HMBC correlations of compounds **1** and **2**.

Compound **2** was previously isolated from *Salvinia molesta* D.S. Mitchell (Salviniaceae), and the spectroscopic information, as well as the optical rotation, were consistent with the reported values.^27,28^ The known compounds crocinervolide (**3**), emodin (**4**), erythrolaccin (**5**), and laccaic acid B (**6**) were characterized by comparing the spectral properties with those previously reported.^29–32^ Finally, laccaic acid E (**7**), xantholaccaic acid B (**8**), laccaic acid A (**9**), laccaic acid monomethyl ether (**10**), and an isomer of laccaic acid B (**11**) (Table 2) were identified using UPLC/MS/MS analyses.^11,13^ Therefore, the lac produced by *T. fulgens* biosynthesizes laccaic acids similar to *K. lacca*. However, unlike the latter, it does not contain α-cedrene and *R*-curcumene acid derivatives^33^ instead, it includes conjugated farnesol and nerolidol acid derivatives with one or two hydroxyl functional groups. It is important to note that the anthraquinones of arí are medicinally active, as Kabeer et al.^34,35^ demonstrated that a mixture of laccaic acids reduced insulin resistance in C57BL/6J mice, decreased the expression of inflammatory cytokines, and lowered gluconeogenesis via KDM2A mediated by MicroRNA-721. On the other hand, emodin improves insulin sensitivity and β-cell function by influencing multiple molecular pathways. This anthraquinone exhibits a range of other biological activities, including antioxidant, antimicrobial, immunomodulator, hepatoprotective, and laxative properties.^36^

**Table 2:** UHPLC/UV-MS Data of Compounds Tentatively Detected in Fraction **F2** of **IA** from Sonora Gum^a^

compound	molecular formula	RT (min)	UV–vis (nm)	experimental [M – H]^−^(*m*/*z*)	Δ (ppm)	du
**7**	C_24_H_17_NO_11_	1.14	218, 282, 486sh	494.07315	0.5	17
**8**	C_24_H_16_O_11_	2.33	225, 287, 487sh	479.06195	–0.1	17
**6** ^b^	C_24_H_16_O_12_	2.46	223, 287, 487sh	495.05670	–0.4	17
**9**	C_26_H_19_NO_12_	2.49	223, 287, 487sh	536.08307	–0.7	18
**10**	C_27_H_21_NO_12_	2.79	220, 287, 489sh	550.09918	0.1	18
**11**	C_24_H_16_O_12_	3.24	223, 287, 486sh	495.05707	–0.4	17

a sh: shoulder band in UV–vis spectrum; du: degree of unsaturation.

b Identified with coelution.

### 3.2 Volatilome from Sonora Gum

Analyzing the volatilome in arí is important for authentication and sensory quality as the volatile components significantly influence the perception of taste in foods and beverages. To determine the arí’s volatilome, headspace-solid phase microextraction analysis was performed using four different coated fibers composed of polydimethylsiloxane (PDMS), divinylbenzene (DVB), or carboxen (CAR). The extraction was coupled to GC-MS analysis, and according to the total ion chromatograms, the less polar PDMS fiber allowed the identification of a higher number of components. Sixty-one compounds were identified (Table S1, Figures S17 and S18). The compounds characterized were divided into four groups: lilac analogs (linalool derivatives), a few cedrene alcohol derivatives, and a few fatty acid derivatives, particularly myristic acid. The fourth group consisted of no identified compounds (Figure 4). Interestingly, lilac derivatives, although present in low concentrations, may contribute to the sensory properties of arí, similar to their effects in orange and rosemary honey, which are known for their sweet flavor and slight bitterness.^37^ In contrast, cedrene alcohol derivatives were identified as the main sesquiterpenoids, which is unexpected since no volatile cedrene derivatives are typically found in shellac.^38^ Unfortunately, no studies on the volatilome of *K. lacca* exist to indicate the potential presence of volatile cedrene derivatives. Regarding myristic acid, its high detected proportion suggests its significance in the gum’s hydrophobicity. For instance, in *K. lacca*, the shellac resin consists of long-chain aliphatic hydroxy acids (aleuritic acid, mainly) linked to cyclic sesquiterpene acids, forming a unique nanostructure with hydrophilic and lipophilic phases that give rise to an amphiphilic structure at the interface.^38^

**Figure 4:**
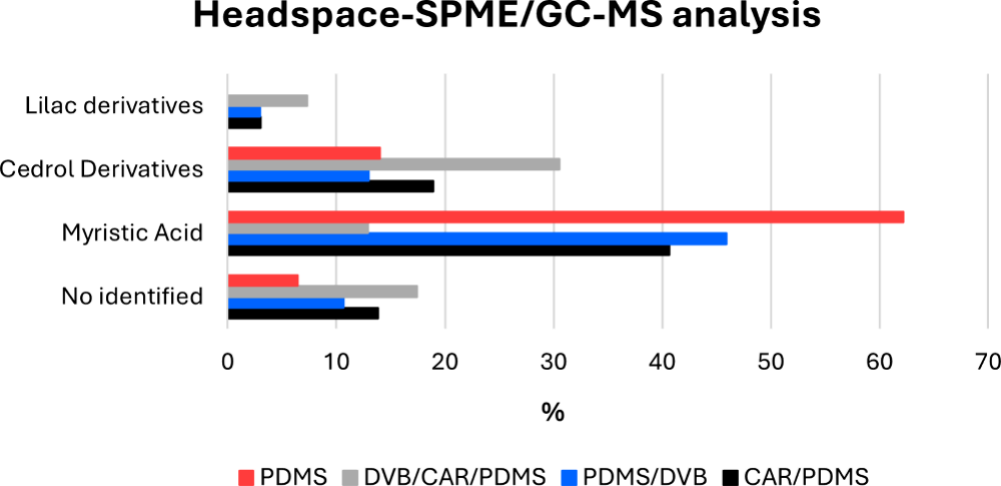
Families of compounds identified in Sonora gum using HS-SPME/GC-MS.

### 3.3 Isolation of **12**–**15** from *C. glandulosa*

For comparative purposes, an acetonic and an aqueous extract of the rosary babybonnets, the host species of *T. fulgens*, were also chemically analyzed. The most relevant compounds isolated were (+)-pinitol (**12**),^39,40^ stigmasterol (**13**), β-d-glucositosterol (**14**), and ursolic acid (**15**),^41^ which were identified based on their physical and spectroscopic data. According to TLC and NMR analyses, the aqueous extract also contained a considerable amount of (+)-pinitol. Interestingly, (+)-pinitol is an effective supplement for treating type II diabetes mellitus. Most animal and clinical studies have demonstrated that this polyalcohol regulates hyperglycemia and prevents insulin resistance.^42^

### 3.4 Nutritional Value of Sonora Gum

A proximal analysis was conducted to gain insight into arí’s nutritional value because it provides information about the primary nutrients in food. The official international methods of the Association of Official Analytical Chemists (AOAC) were employed.^17^ This proximate analysis of arí involved assessing key components, such as moisture, ash, crude protein, fat, crude fiber, minerals, fatty acids, amino acids, and carbohydrates.

The content of moisture, fat, protein, carbohydrates, ash, and overall energy (200 kcal) is low (Table 3), although insects, according to recent studies, are a good source of protein (30–70%) and lipids (10–50%).^43–46^ The crude fiber content, however, is high. Fiber plays an important role in increased nitrogen utilization and the absorption of some other micronutrients. Fibers are also effective for lowering serum cholesterol and constipation, among other benefits. Thus, arí can be considered to be a valuable source of dietary fiber in human nutrition.

**Table 3:** Proximate Composition, Vitamins, and Mineral Content of Sonora Gum

	value^a^
Proximate Composition^b^	
moisture	0.49 ± 0.02
ash	0.94 ± 0.01
total fat (ethereal extract)	3.45 ± 0.11
protein	14.67 ± 0.16
crude fiber	52.82 ± 2.57
carbohydrates	27.63
energy (kcal/100 g)	200.25
Vitamins^c^	
vitamin C	0.18 ± 0.01
niacin	16.24 ± 0.66
thiamin	9.01 ± 0.45
riboflavin	0.80 ± 0.01
folic acid (μg)^d^	6.26 ± 0.26
Minerals^c^	
Na	31.73 ± 1.48
K	107.67 ± 3.94
Ca	75.22 ± 1.29
Fe	3.86 ± 0.13
Mg	29.90 ± 1.31
Cu	1.62 ± 0.08
Zn	1.43 ± 0.06
P	226.07 ± 10.5

a Mean ± standard deviation, *n* = 3, RSD < 5%.

b g/100 g dry weight.

c mg/100 g dry weight.

d Quantified as folic acid.

The analysis of microelements (Table 3) revealed that arí is not rich in minerals. Phosphorus was the more relevant element, but its amount was much lower than its content in phosphorus-rich foods such as milk and nuts.^46^ Arí was rich in vitamins B3 and B1 as are edible grasshopper species *Ruspolia differens* Serville and *Brachystola magna* Girard. Vitamin B3 or niacin is key to synthesizing NAD and NADP which are involved in over 400 biochemical reactions in the body;^47^ it keeps the skin, hair, and nervous system healthy. Thiamine (B1) plays a unique role in the metabolism of carbohydrates, fats, and proteins, cellular respiration, and the oxidation of fatty acids. It ensures the functioning of the nervous system.

The amino acid analysis revealed a high content of serine and histidine (Table 4); the latter, an essential amino acid, promotes digestive health and histamine regulation, among other actions; serine, on the other hand, is a brain booster. Serine and histidine were present in higher amounts than other edible insects, such as the Bombay locust, scarab beetle, house cricket, and mulberry silkworm.^48,49^

**Table 4:** Content of Principal Amino Acids in Sonora Gum

amino acids	value^a^
aspartic acid	0.48
serine	4.40
histidine	4.57
threonine	0.44
tyrosine	0.34
methionine	0.25
valine	0.35
leucine	0.30
isoleucine	0.24
lysine	0.35

a g/100 g dry weight, *n* = 3, RSD < 5%.

The fatty acid content was evaluated in detail (Table 5); the determination involves hydrolytic extraction followed by gas chromatography to measure fatty acid methyl esters (FAMEs). Eleven fatty acids were detected. Myristic acid is the main saturated acid, confirming the headspace-solid phase microextraction analysis results. Monounsaturated fatty acids were also present, and the only polyunsaturated fatty acid was linoleic acid, an omega six fatty acid.

**Table 5:** Content of Fatty Acids in Sonora Gum

fatty acid	value^a^
lauric, 12:0	0.03
myristic, 14:0	1.92
myristoleic, 14:1	1.34
palmitic, 16:0	0.15
palmitoleic, 16:1	0.06
margaroleic, 17:1	0.02
stearic, 18:0	0.05
oleic, 18:1	0.35
linoleic, 18:2	0.02
erucic, 22:1	0.07

a g/100 g dry weight, *n* = 3, RSD < 5%.

Finally, the composition of polyalcohols was assessed because the host plant is rich in (+)-pinitol. However, arí does not contain (+)-pinitol or any other cyclitol.

### 3.5 Potential Acute Toxicity and Antihyperglycemic Effects of the Arí Aqueous Extract

The acute toxicity was analyzed using the Lorke procedure (Table S2), which was one of the first tests conducted prior to further toxicity analyses. Its primary goal is to establish signs of toxicity and death. The Lorke method involves two phases. In the first phase, nine animals were divided into three groups and given 10, 100, and 1000 mg/kg body weight of IA to establish the dose range that produces any toxic effects. In the second phase, three geometric doses of IA were administered according to the established protocol (1600, 2900, and 5000 mg/kg), based on the results of the first phase. In this case, no mortality or internal damage was observed. Therefore, the LD_50_ was estimated to be higher than 5 g/kg, indicating that the extract is practically nontoxic to mice.

Given the high prevalence of diabetes in Mexico and the presence of laccaic acids and emodin in arí, as well as the indiscriminate use of natural products, including arí, for diabetes treatment, the antihyperglycemic effect was evaluated by using a glucose tolerance test. As shown in Figure 5, IA significantly reduced the postprandial peak at the highest dose tested (316 mg/kg) in healthy mice. In hyperglycemic mice, the effect was more pronounced at this highest dose (316 mg/kg) and remained comparable throughout the entire evaluated time course to that of the standard drug MET. While these results do not yet propose a mechanism of action, they indicate that arí improves glucose utilization in both healthy and hyperglycemic states. Several mechanisms may contribute to enhancing glucose utilization. The antihyperglycemic action observed may be attributed to the presence of laccaic acid derivatives and emodin, which improve insulin resistance.^35,36^ However, the involvement of other components of arí cannot be dismissed and warrants further investigation.

**Figure 5:**
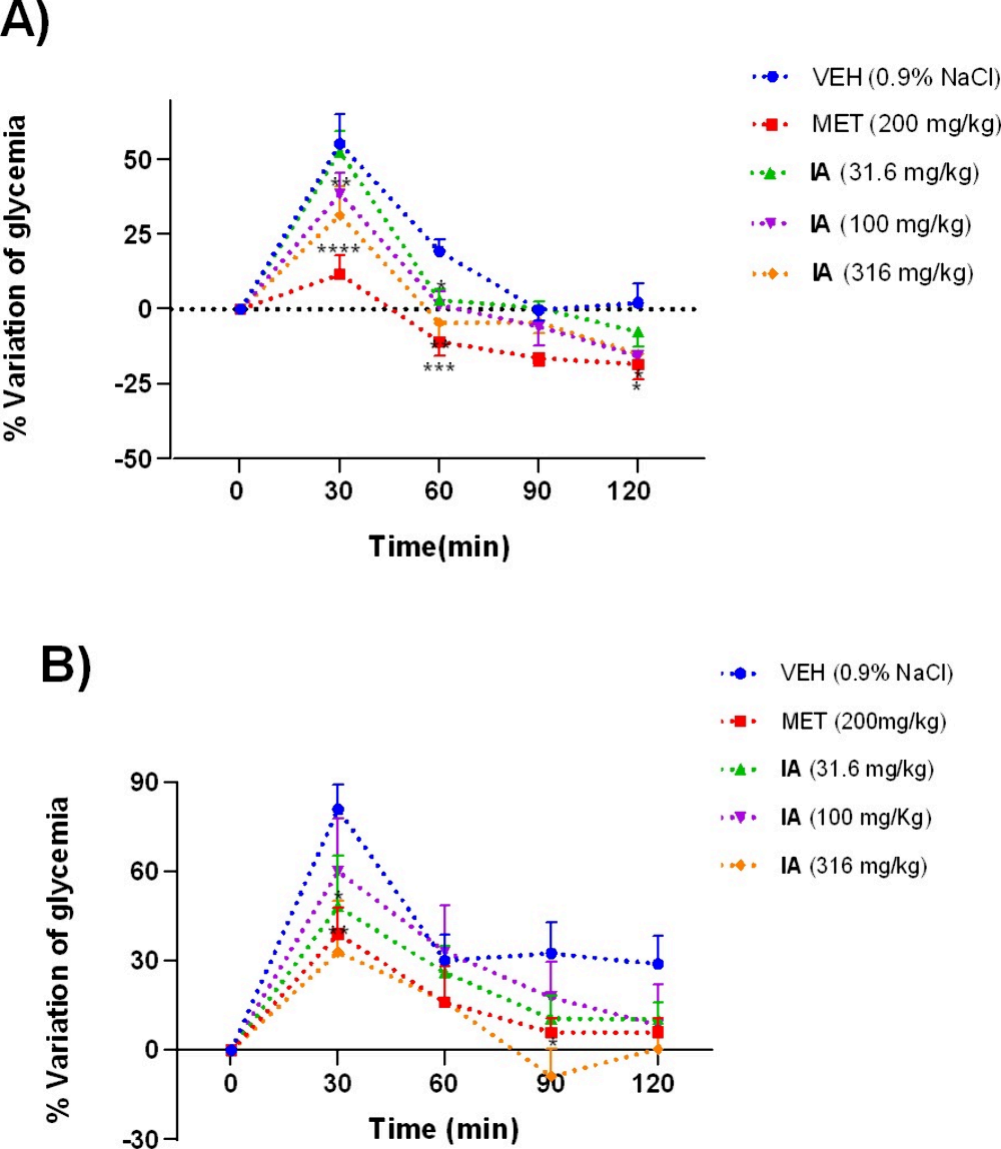
Time course of the OGTT of IA in (A) normoglycemic and (B) hyperglycemic mice. VEH: 0.9% NaCl, MET: metformin. Each measurement is presented as the mean ± SEM, with six mice per group. Significantly different from VEH (*p < 0.05, **p < 0.01, ***p < 0.001, and ****p < 0.0001), as determined by two-way ANOVA followed by Dunnett’s post hoc test.

## 4 Conclusions

This study was conducted to explore the nutritional and antidiabetic properties of arí, emphasizing its traditional value as both food and medicine. The proximal analysis and chemical composition demonstrated that arí contains a significant amount of fiber and vitamins B1 and B3. Similar to *K. lacca* resin, arí from *T. fulgens* contains anthraquinones with potential antidiabetic metabolites. Compared to *K. lacca*, whose terpenoids acids are derived from α-cedrene and *R*-curcumene acids, those found in arí originate from farnesol and nerolidol acids. Regarding the observed antihyperglycemic effect, this could be attributed to the presence of anthraquinones, specifically emodin and laccaic acids, which have demonstrated antidiabetic activity. However, further studies are required to determine the extent to which the isolated sesquiterpenes and other detected metabolites may influence this effect. In any case, this work provides evidence of the current use of arí as an antidiabetic agent. Additionally, it was demonstrated that the host plant of *T. fulgens* has a different composition and shows promise as an antidiabetic agent. These results will support the rational use of Sonora gum and enhance the understanding of Mexican ancestral foods and medicines.

## Supplementary Material



## Notes

The NMR spectra for compounds **1** and **2** (Figures S1–S16). Total ion current chromatograms of the volatile components from Sonora gum using HS–SPME (Figure S17). Mass spectra of volatile compounds (Figure S18). Identified volatile compounds from Sonora gum by HS-SPME/GC-MS (Table S1). Lorke assay data (Table S2). Process of extraction and pharmacological analysis of the arí (Figure S19). Process of extraction of *C. glandulosa* (Figure S20) (PDF)

## Notes

Dedicated to Professor Dr. Iklas Khan, National Center for Natural Products Research, University of Mississippi, on the occasion of his 65th birthday.
