# Supplementary material for: Chemical, Nutritional, and Antihyperglycemic Studies on Sonora Gum
Source: ACS Omega. 2025 Jun 18;10(25):26964–74. doi: 10.1021/acsomega.5c02073 (PMC12223910; doi:10.1021/acsomega.5c02073)
Supplement: Supplementary file 1 [file ao5c02073_si_001.pdf]

Supplementary Material

to the article

Chemical, nutritional, and antihyperglycemic studies on Sonora gum#

Araceli Pérez-Vásquez[a], Vanya Meneses-Pérez[a], Valeria Reyes-Pérez[b], Laura Flores-Bocanegra[a], Manuel Rangel Grimaldo[b], Edelmira Linares[c], Robert Bye[c,*], Rachel Mata[a,**]

[a] Facultad de Química, Universidad Nacional Autónoma de México, Ciudad de México 04510

[b] Instituto de Química, Universidad Nacional Autónoma de México, Ciudad de México 04510

[c] Instituto de Biología, Universidad Nacional Autónoma de México, Ciudad de México 04510

* Corresponding author.

** Corresponding author.

E-mail addresses: rachel@unam.mx, bye.robert@gmail.com.

Dedicated to Professor Dr. Iklas Khan, National Center for Natural Products Research, University of Mississippi, on occasion of his 65th birthday.

Figure S1. HRESIMS (positive mode) spectrum of 1. 3
Figure S2. IR (FTIR-ATR) spectrum of 1. 3
Figure S3. ^{1}H NMR spectrum (400 MHz, CH_3OH-d_4) of 1. 4
Figure S4. ^{13}C NMR spectrum (100 MHz, CH_3OH-d_4) of 1. 4
Figure S5. HSQC spectrum of 1. 5
Figure S6. HMBC spectrum of 1. 5
Figure S7. COSY spectrum of 1. 6
Figure S8. NOESY spectrum of 1. 6
Figure S9. MS spectrum (positive mode) of 2. 7
Figure S10. IR (FTIR-ATR) spectrum of 2. 7
Figure S11. ^{1}H NMR spectrum (400 MHz, CH_3OH-d_4) of 2. 8
Figure S12. ^{13}C NMR spectrum (100 MHz, CH_3OH-d_4) of 2. 8
Figure S13. HSQC spectrum of 2. 9
Figure S14. HMBC spectrum of 2. 9
Figure S15. COSY spectrum of 2. 10
Figure S16. NOESY spectrum of 2. 10
Figure S17. Total ion current chromatograms of the volatile components from Sonora gum using HS–SPME. 11
Figure S18. Mass spectra of volatile compounds 11
Figure S19. Process of extraction and pharmacological analysis of the arí 25
Figure S20. Process of extraction of *C. glandulosa*. 25
Figure S21. Structures of 12 and 15 isolated from *C. glandulosa*. 26
Table S1. Identified volatile compounds from Sonora gum by HS-SPME/GC-MS 27
Table S2. Lorke assay data 29

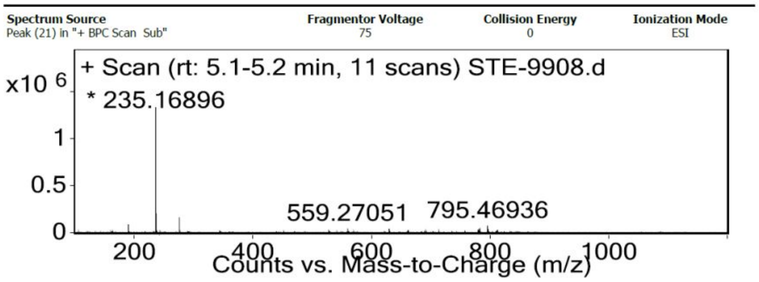

Peak List

m/z	z	Abund
189.16314	1	87431.04
235.16896	1	1334203.18
235.29883		60296.04
236.17191	1	204632.81
275.16068	1	164535.48
559.27051	1	43220.45
629.34532	1	40465.21
781.48331	1	44126.9
795.46936	1	73284.56
796.47234	1	37316.42

Figure S1. HRESIMS (positive mode) spectrum of **1.**

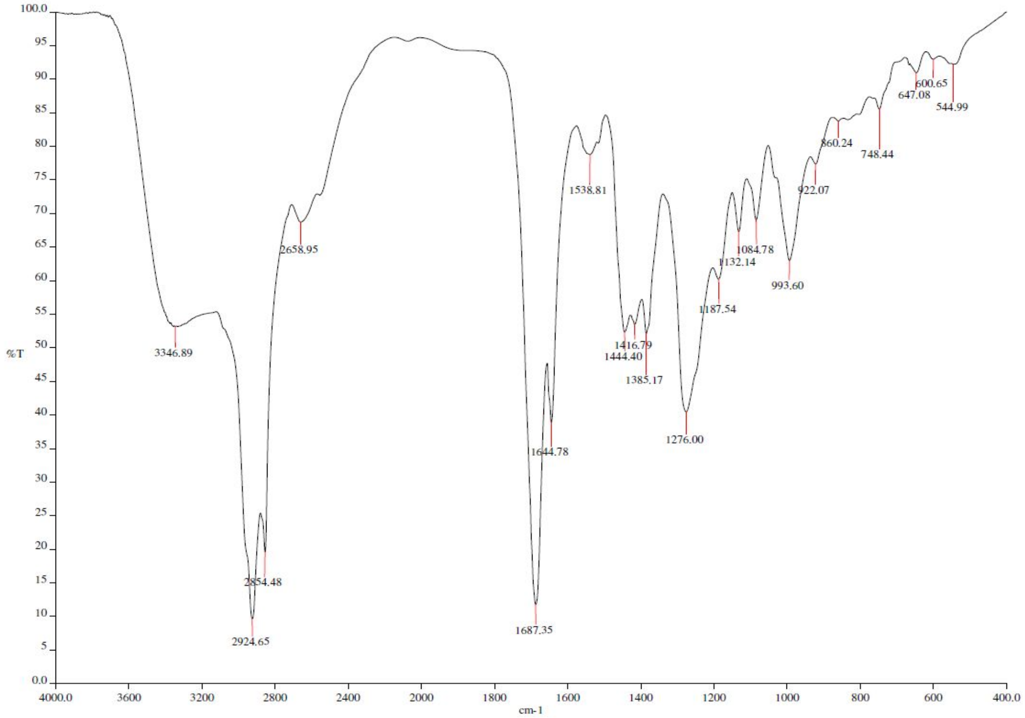

Figure S2. IR (FTIR-ATR) spectrum of **1.**

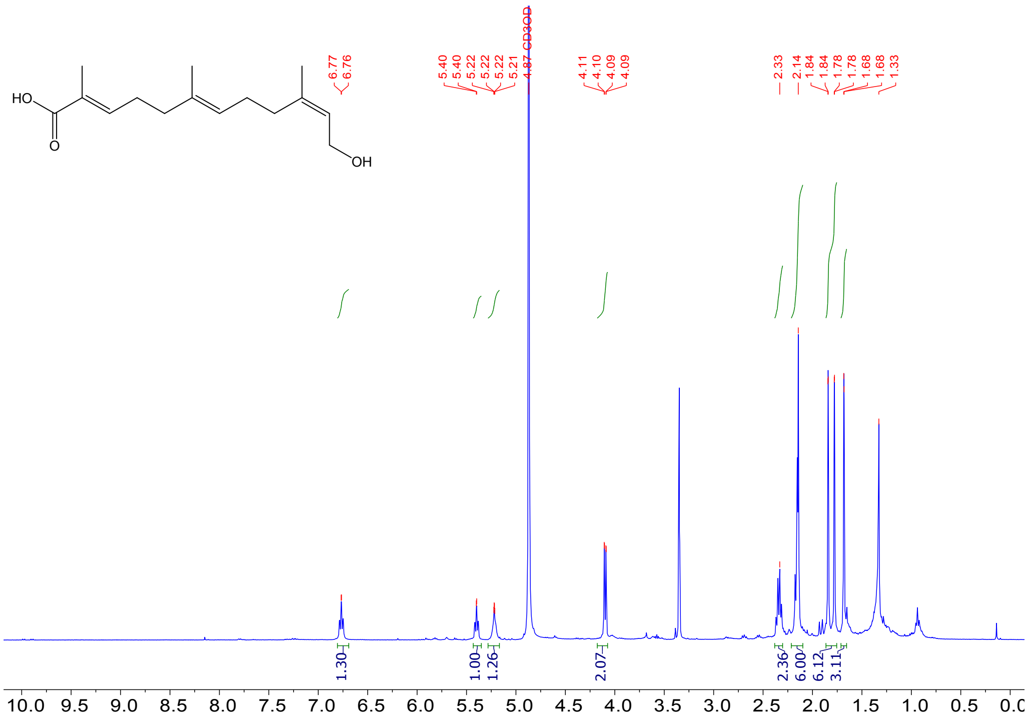

Figure S3. ^{1}H NMR spectrum (400 MHz, CH_3OH-d_4) of **1.**

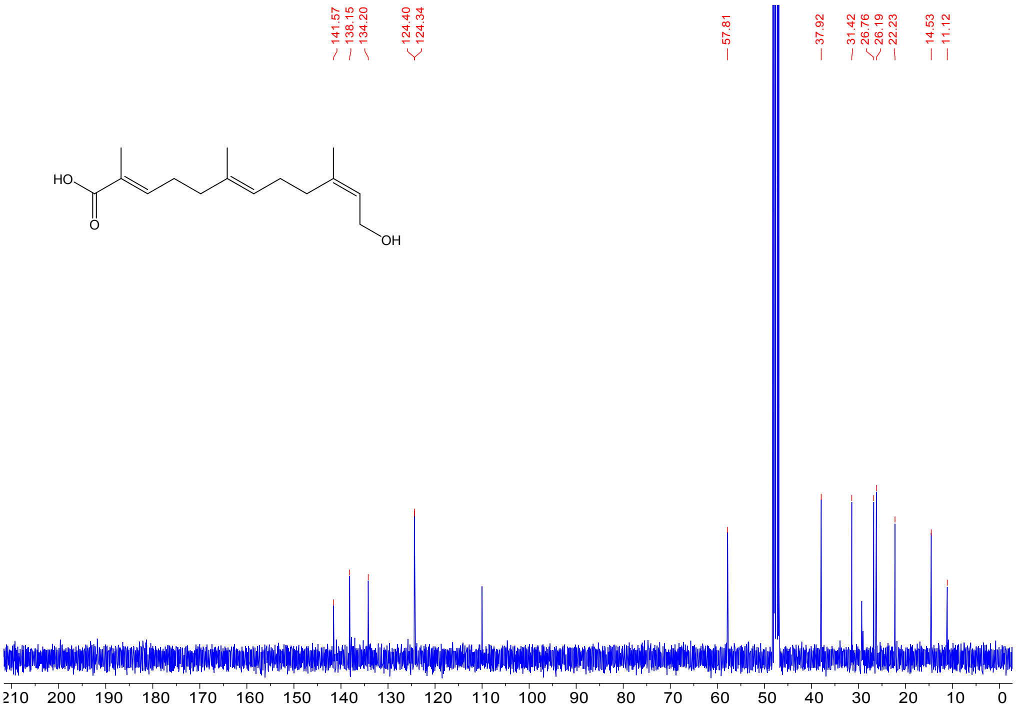

Figure S4. ^{13}C NMR spectrum (100 MHz, CH_3OH-d_4) of **1.**

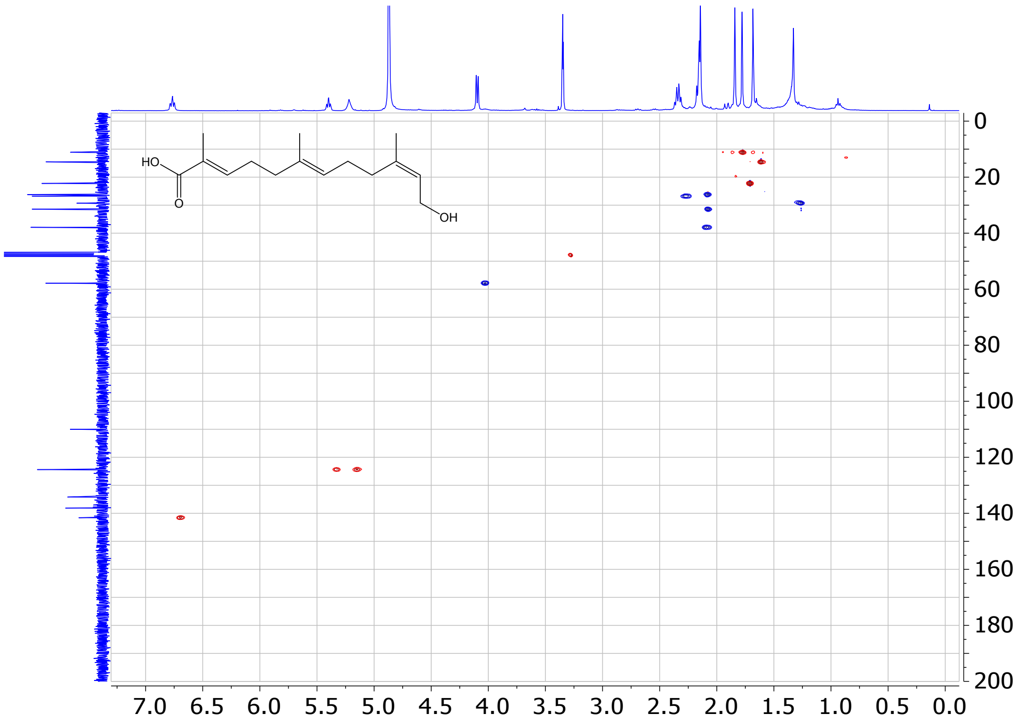

Figure S5. HSQC spectrum of **1.**

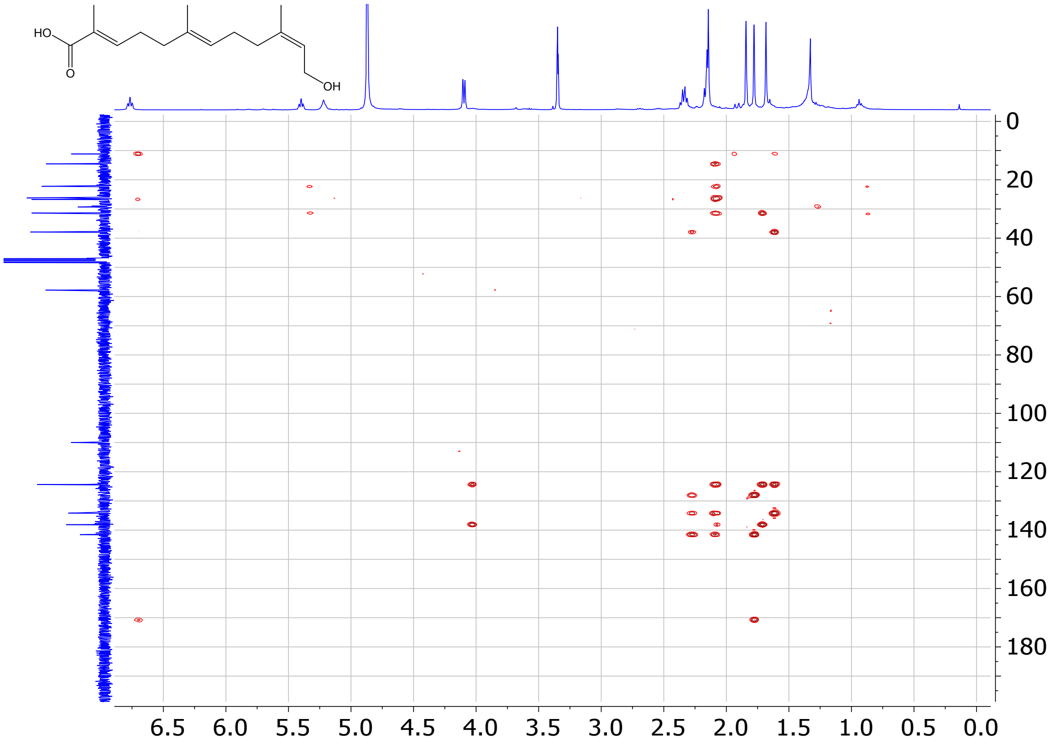

Figure S6. HMBC spectrum of **1.**

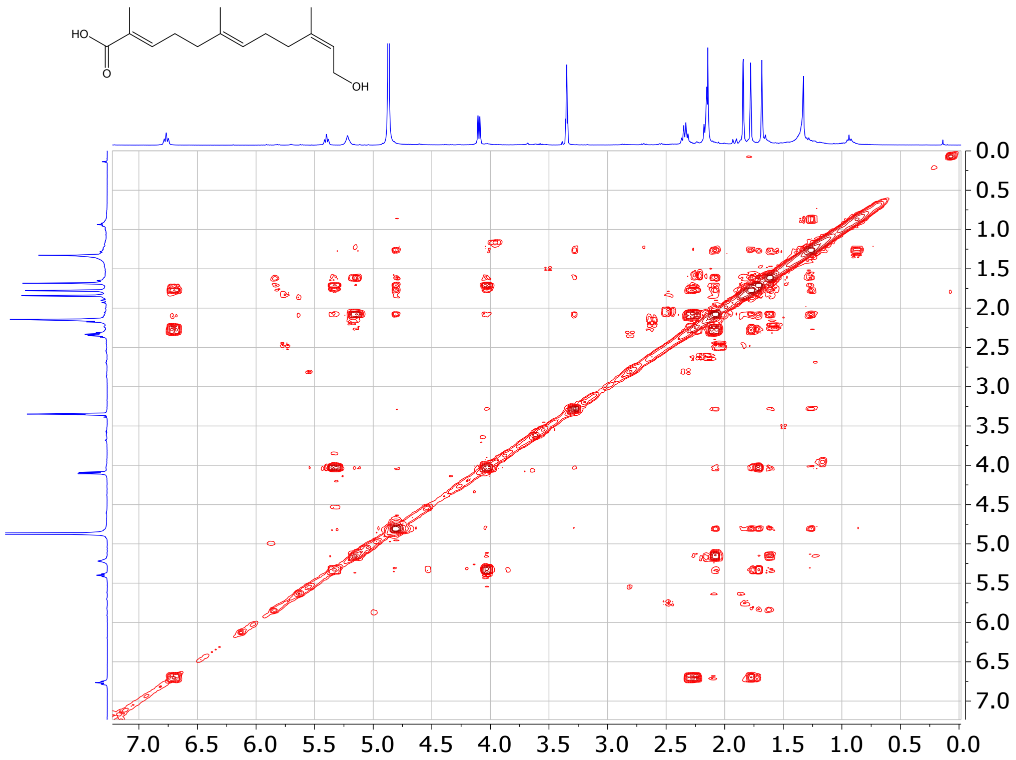

Figure S7. COSY spectrum of **1.**

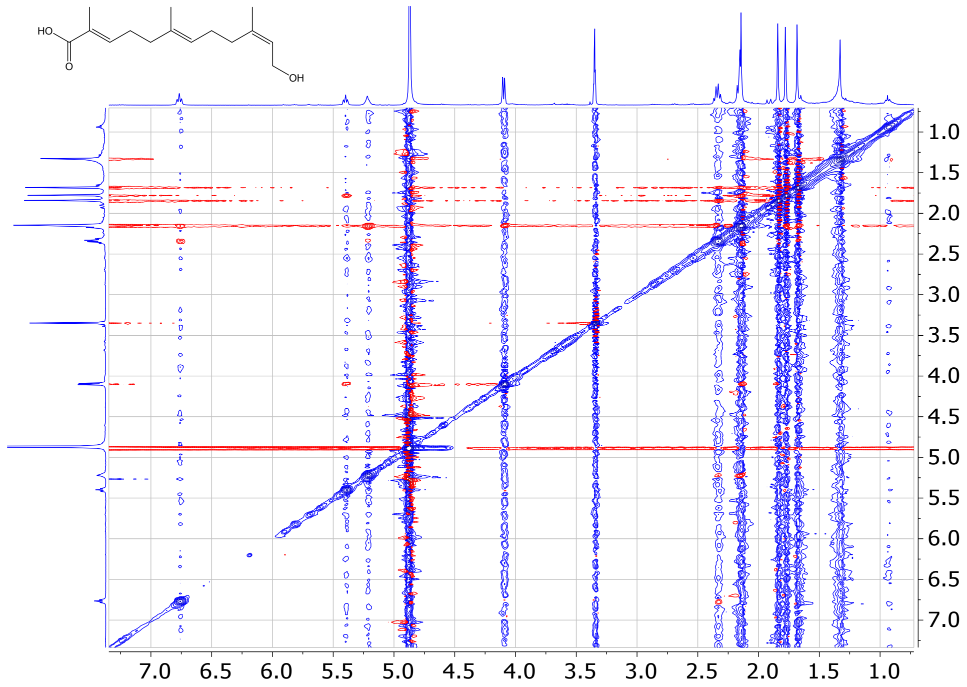

Figure S8. NOESY spectrum of **1.**

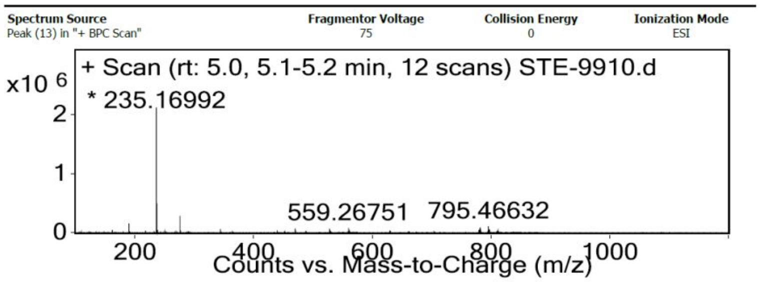

Peak List

m/z	z	Abund
189.16266	1	157595.61
235.16992	1	2131095.05
235.29825		217049.8
236.17121	1	498961.32
275.16002	1	291882.07
343.29305	1	61209.45
527.32587	1	69314.2
559.26751	1	81174.63
781.48033	1	87225
795.46632	1	110916.58

Figure S9. MS spectrum (positive mode) of **2.**

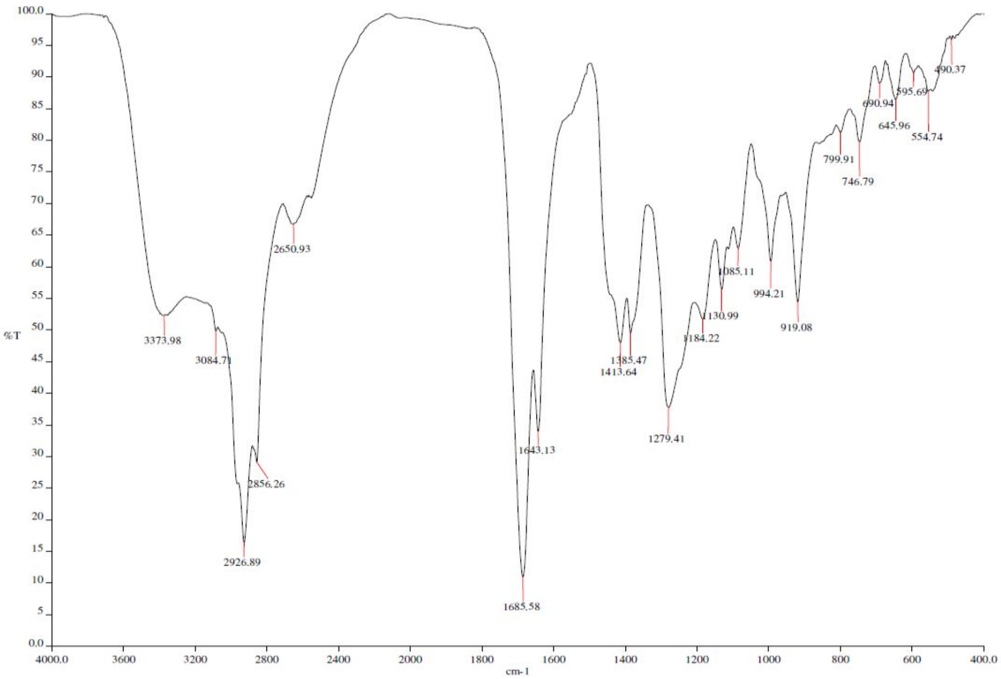

Figure S10. IR (FTIR-ATR) spectrum of **2.**

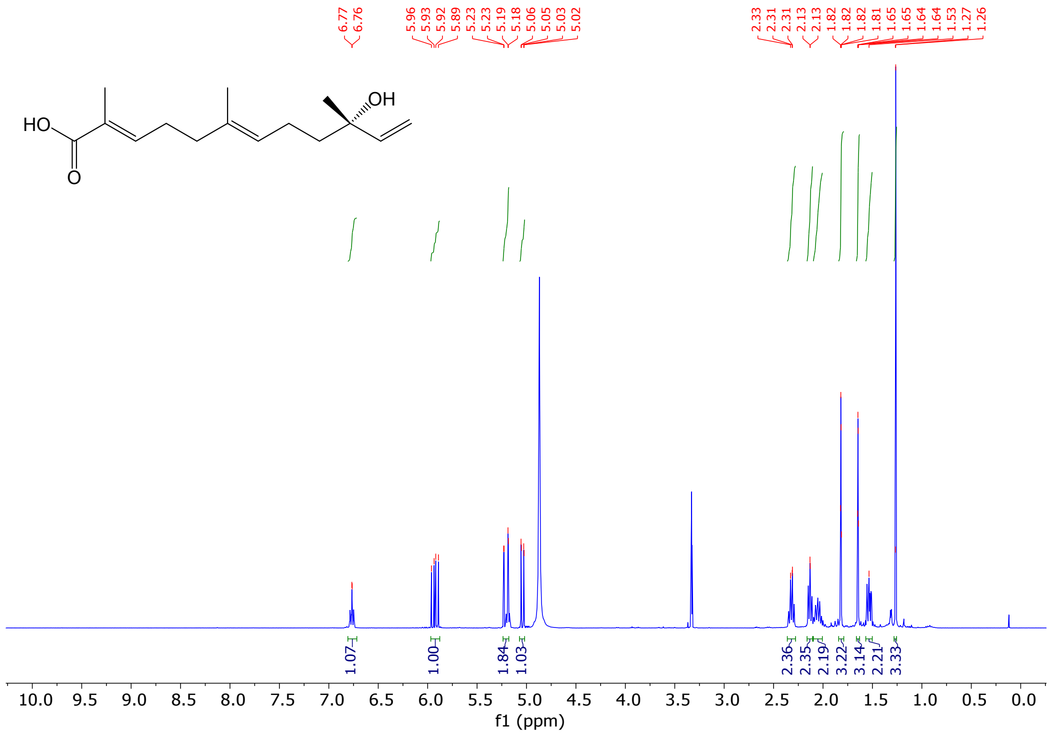

Figure S11. ^{1}H NMR spectrum (400 MHz, CH_3OH-d_4) of **2.**

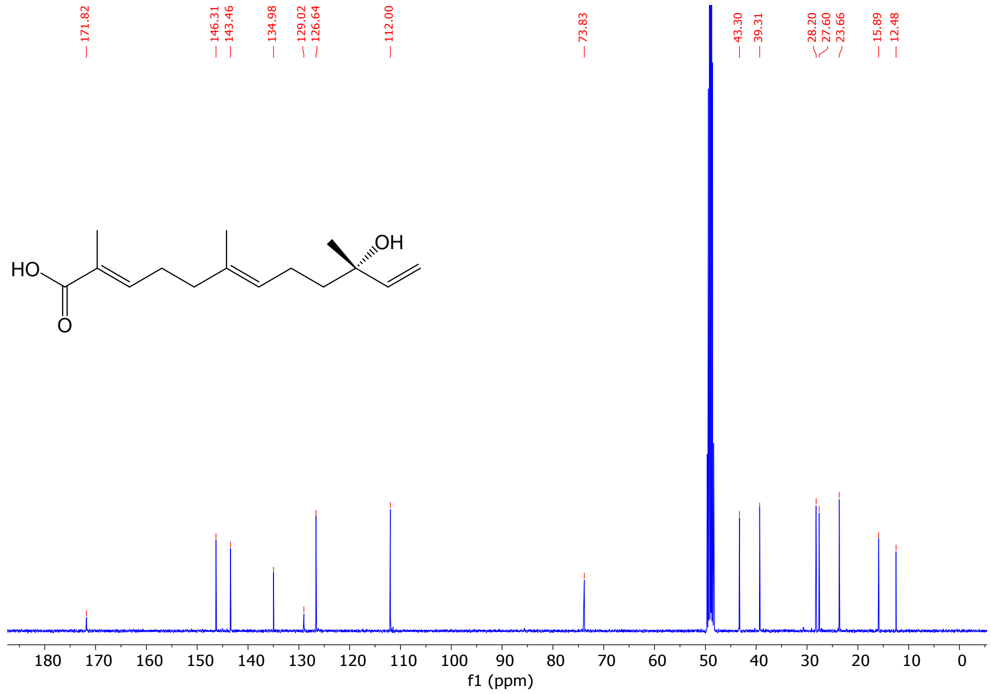

Figure S12. ^{13}C NMR spectrum (100 MHz, CH_3OH-d_4) of **2.**

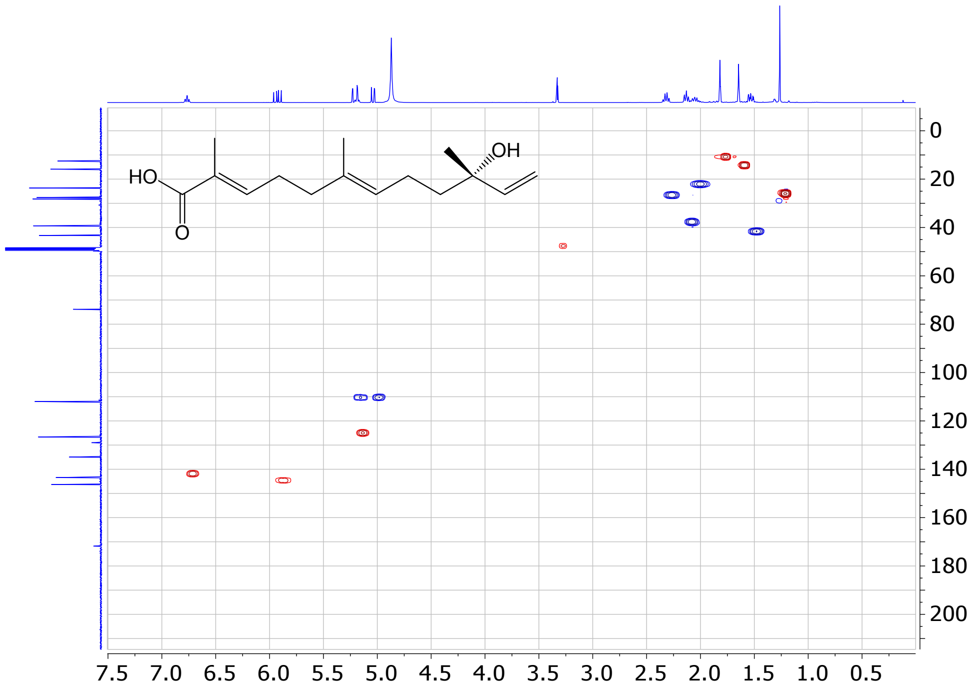

Figure S13. HSQC spectrum of **2.**

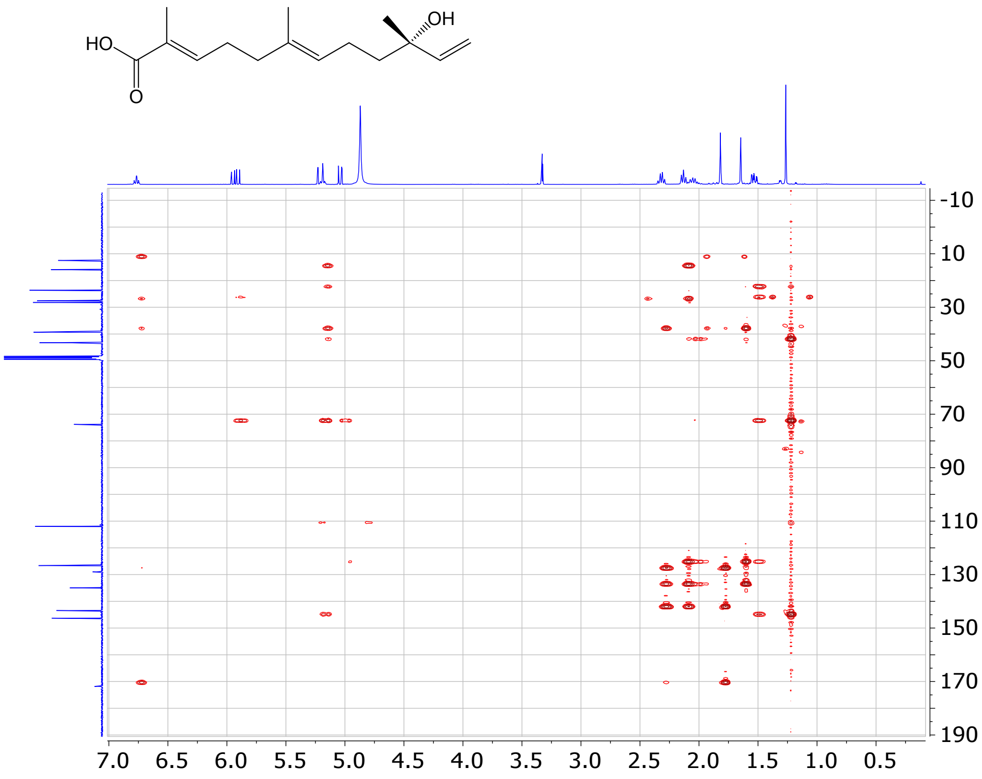

Figure S14. HMBC spectrum of **2.**

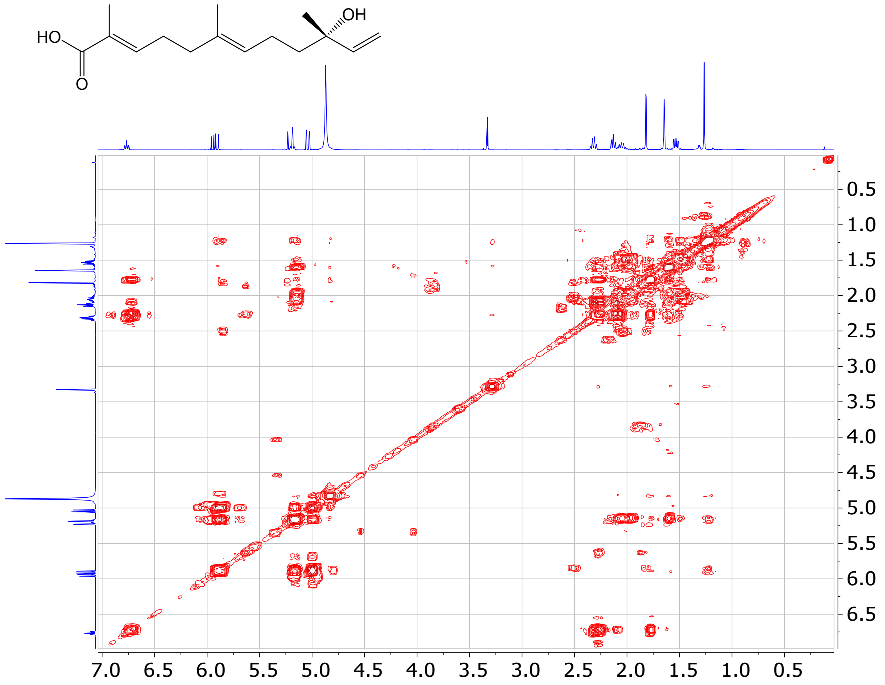

Figure S15. COSY spectrum of **2.**

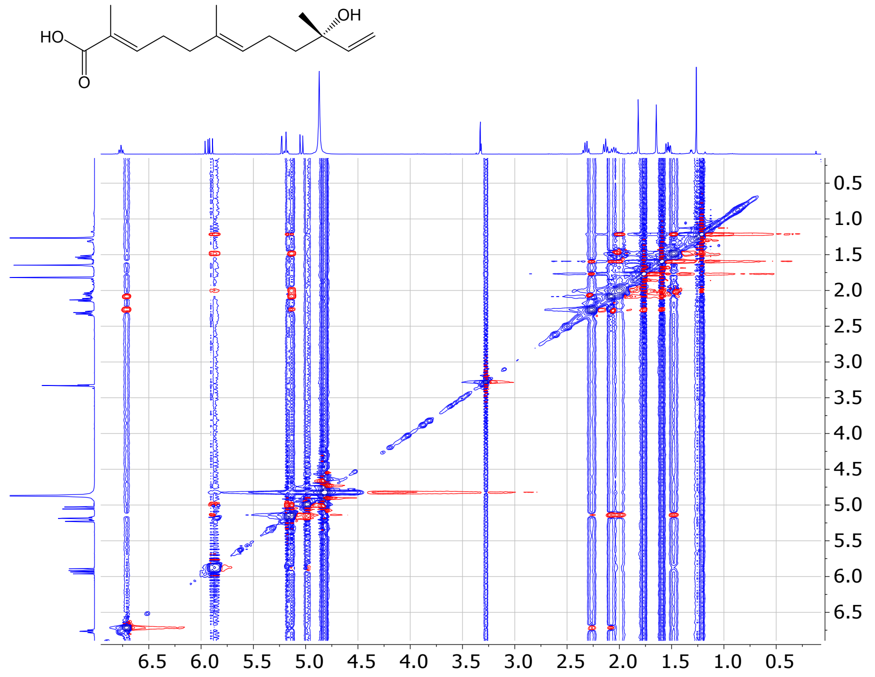

Figure S16. NOESY spectrum of **2.**

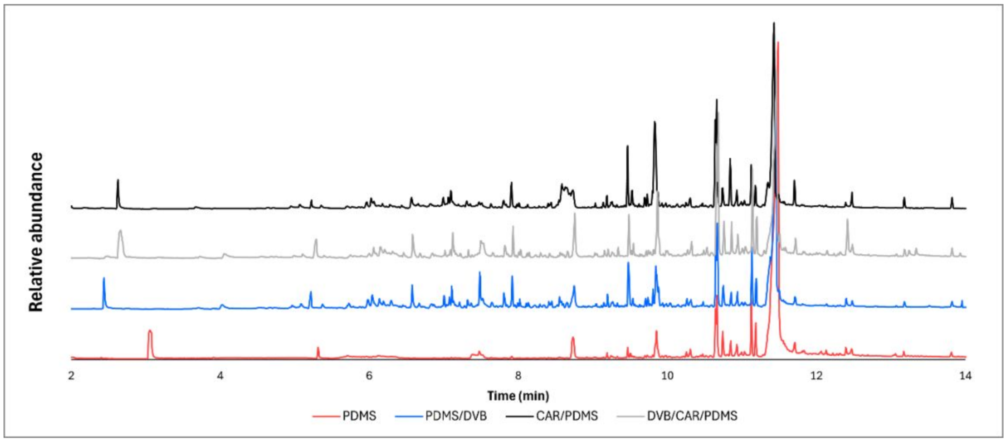

Figure S17. Total ion current chromatograms of the volatile components from Sonora gum using HS–SPME.

Figure S18. Mass spectra of volatile compounds

Methyl amyl ketone (**16**), 4.968 min, (I_r 890)

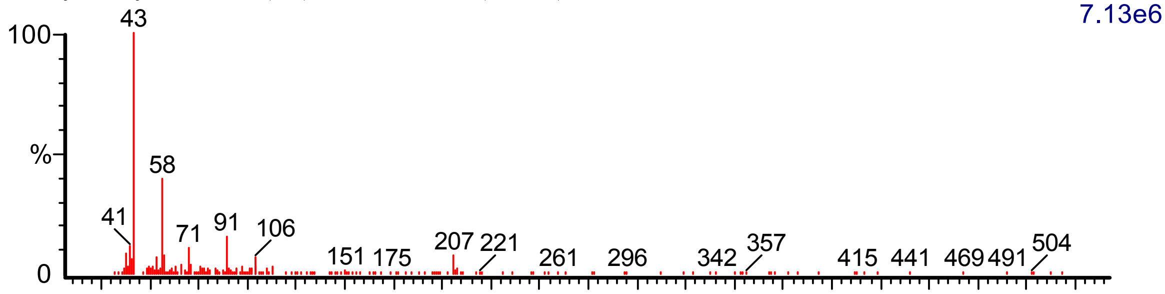

Heptanal (**17**), 5.088 min, (I_r 900)

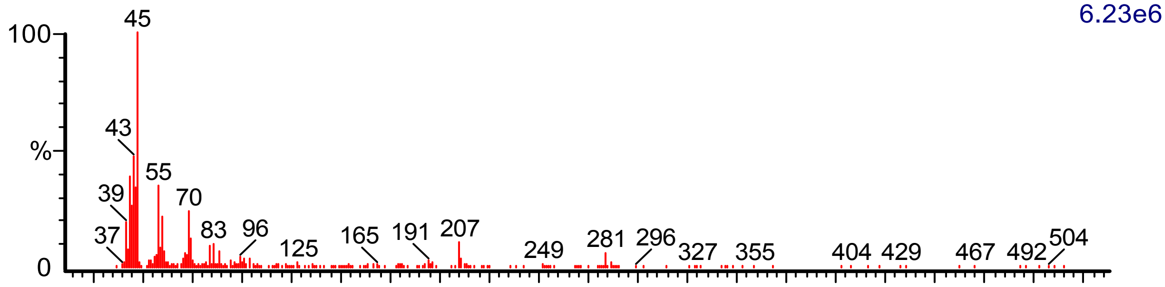

Figure S18. Mass spectra of volatile compounds

Methyl caproate (**19**), 5.373 min, (I_r 924)

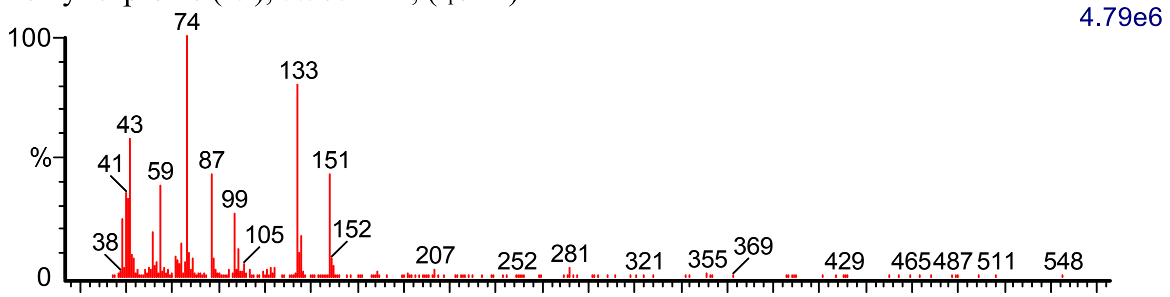

2-Methyl-1-octene-3-yne (**20**), 5.981min, (I_r 978)

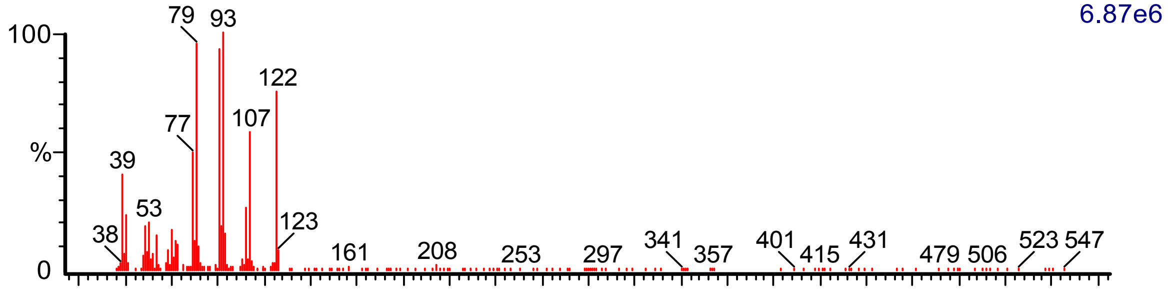

Sulcatone (**21**), 6.026 min, (I_r 985)

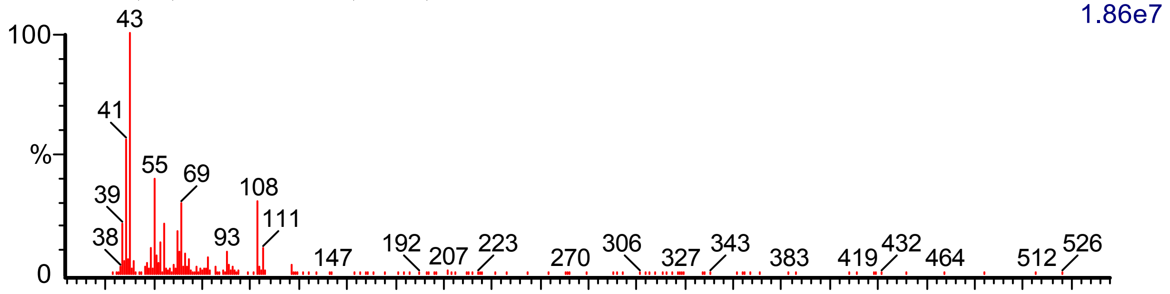

Octanal (**22**), 6.191 min, (I_r 997)

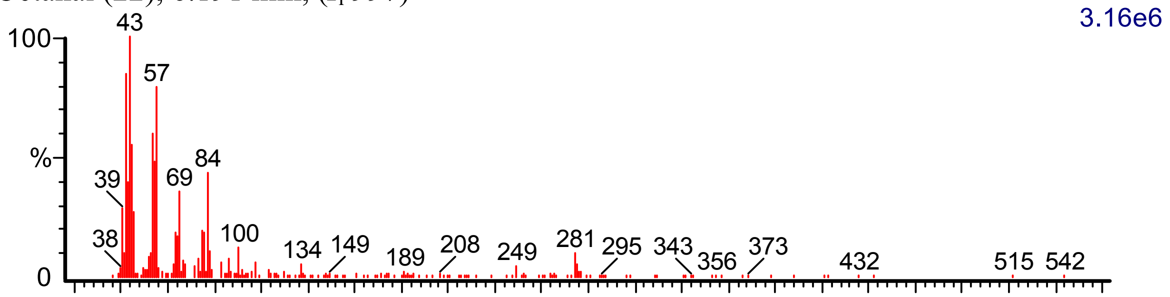

Figure S18. Mass spectra of volatile compounds (continuation)

Lavender lactone (**23**), 6.581 min, (I_r 1038)

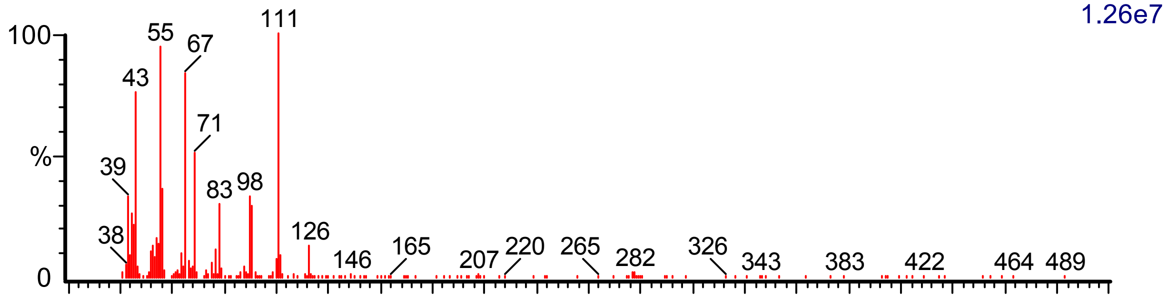

Arbusculone (**24**), 6.656 min, (I_r 1048)

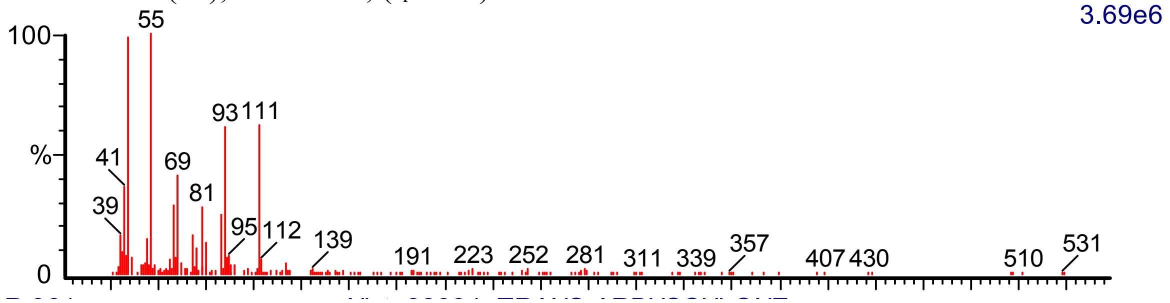

Methyl heptyl ketone (**25**), 7.001 min, (I_r 1084)

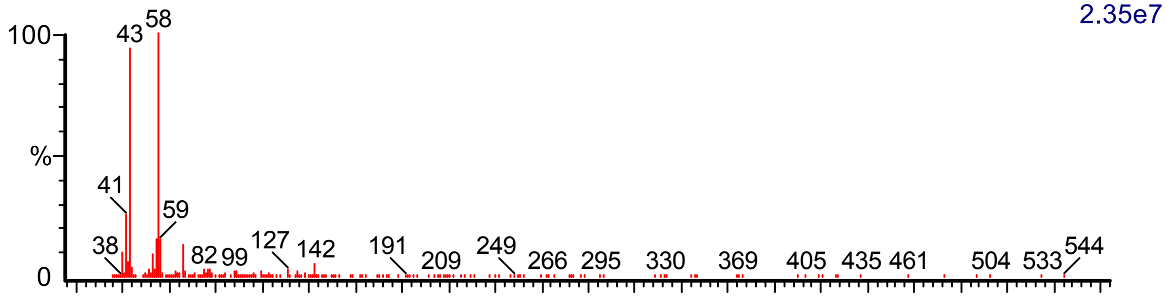

(2)-Nonanol (**26**), 7.061 min, (I_r 1092)

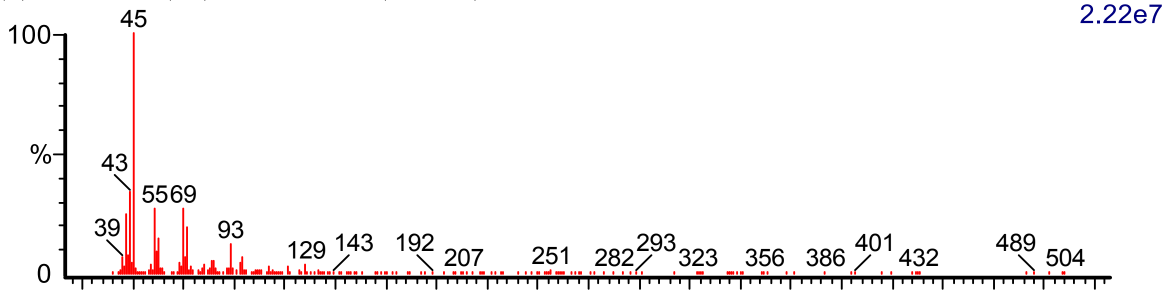

Figure S18. Mass spectra of volatile compounds (continuation)

Nonanal (**27**), 7.091 min, (I_r 1095)

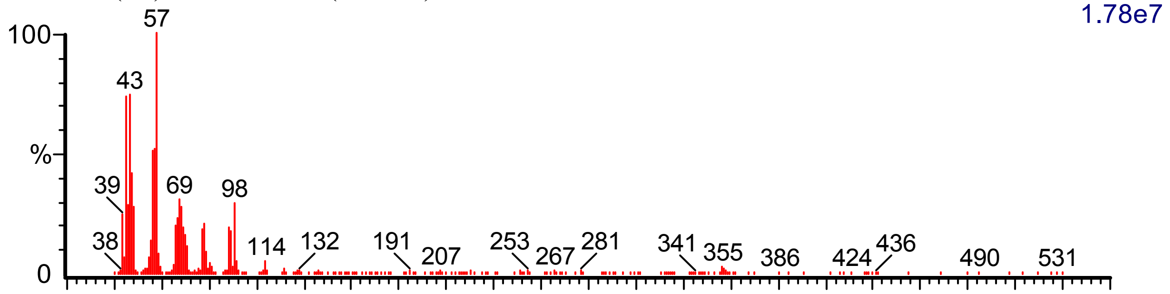

Limona ketone (**28**), 7.369 min, (I_r 1127)

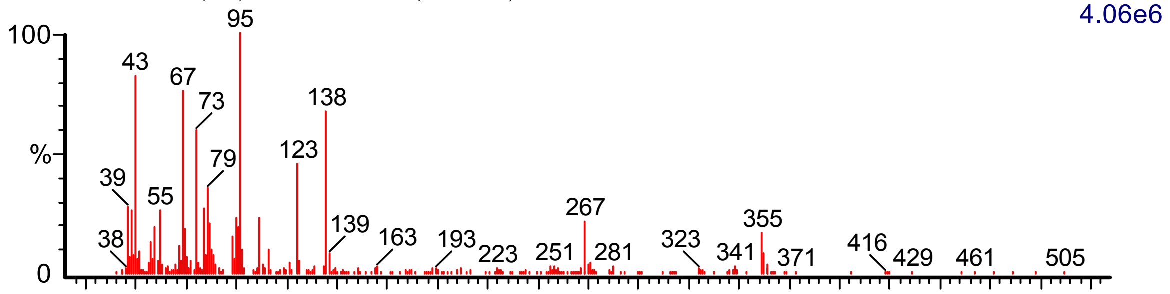

p-Acetotoluene (**29**), 7.811 min, (I_r 1182)

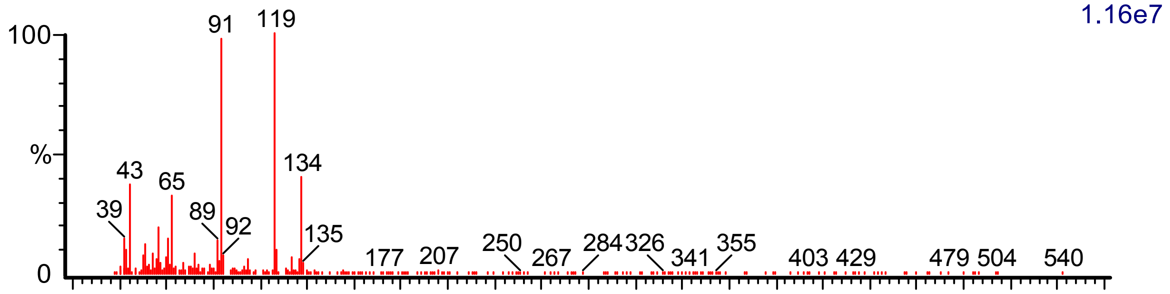

Lilac Alcohol Isomer (**31**), 7.984 min, (I_r 1205)

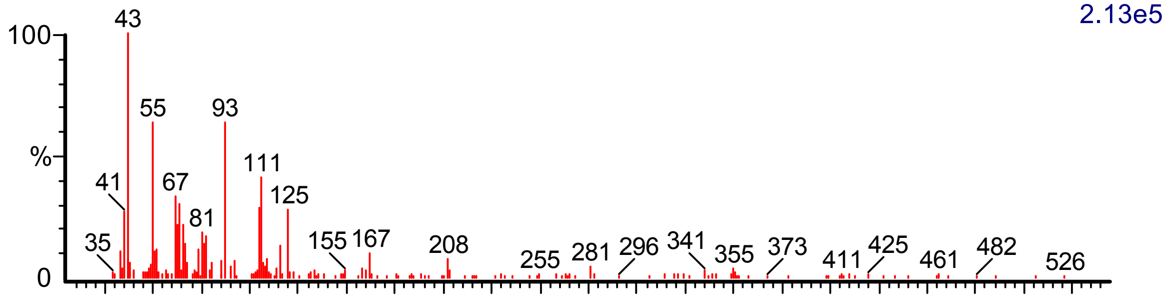

Figure S18. Mass spectra of volatile compounds (continuation)

Lilac Alcohol Isomer (**32**), 8.014 min, (I_r 1209)

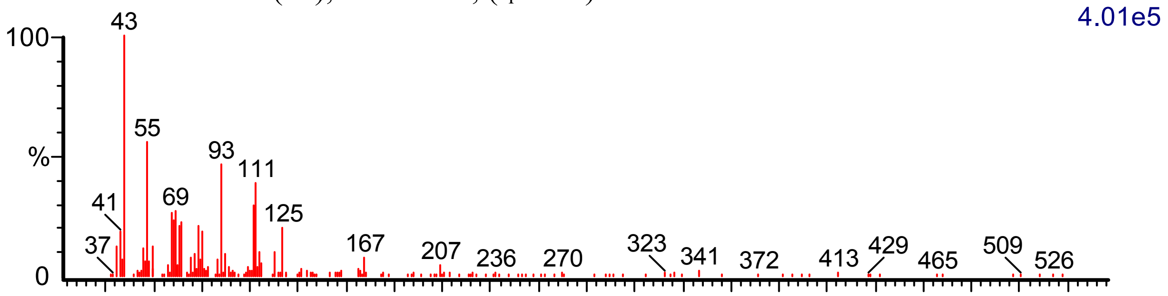

(+)-Nordavanone (**33**), 8.126 min, (I_r 1224)

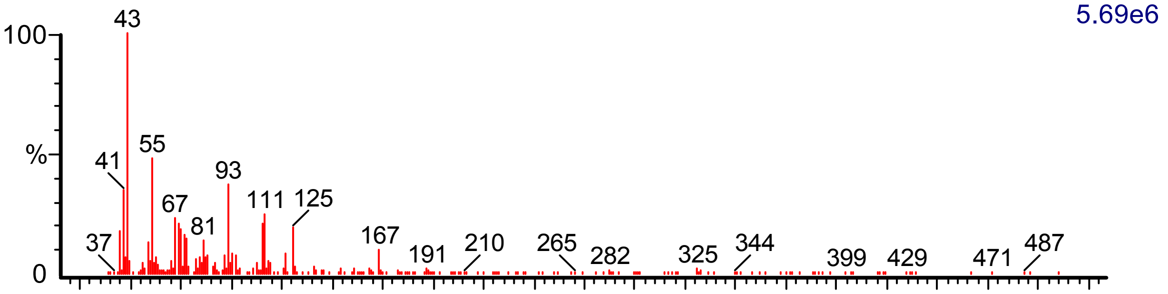

Geraniol (**34**), min, (I_r 1245)

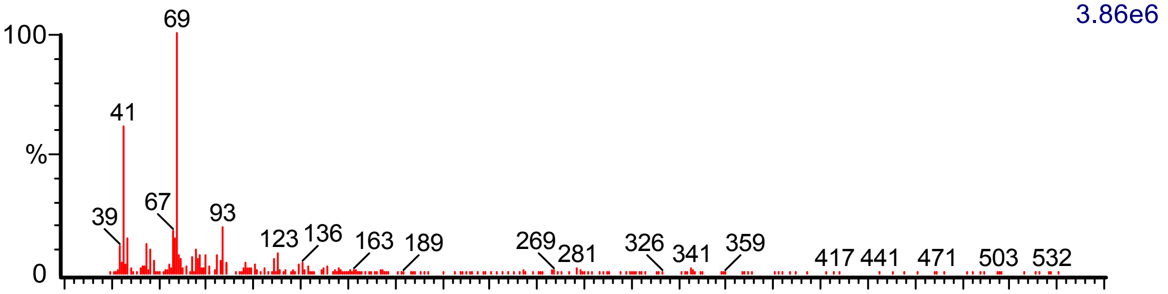

(E)-2-Decenal (**35**), 8.344 min, (I_r 1254)

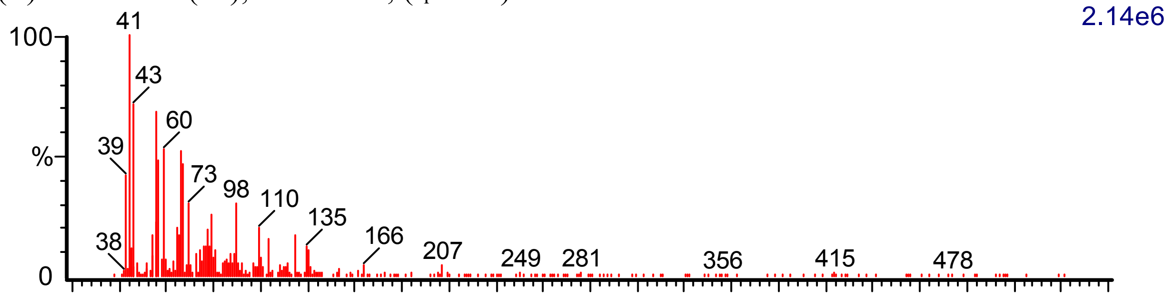

Figure S18. Mass spectra of volatile compounds (continuation)

α-Ionene (**36**), 8.396 min, (I_r 1261)

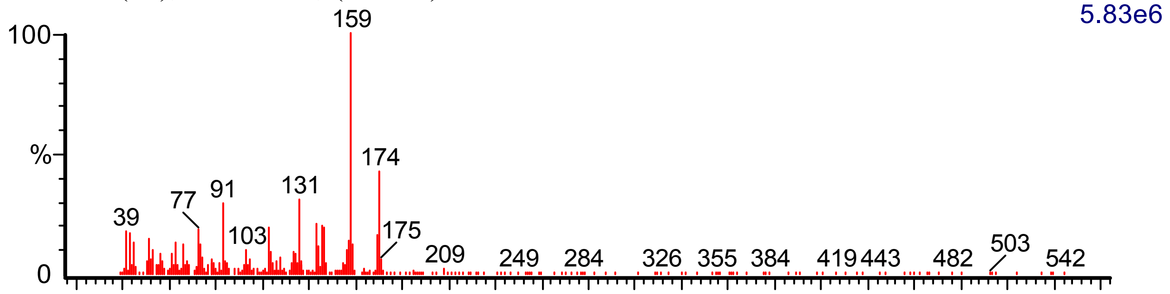

Methyl nonyl ketone (**37**), 8.554 min, (I_r 1289)

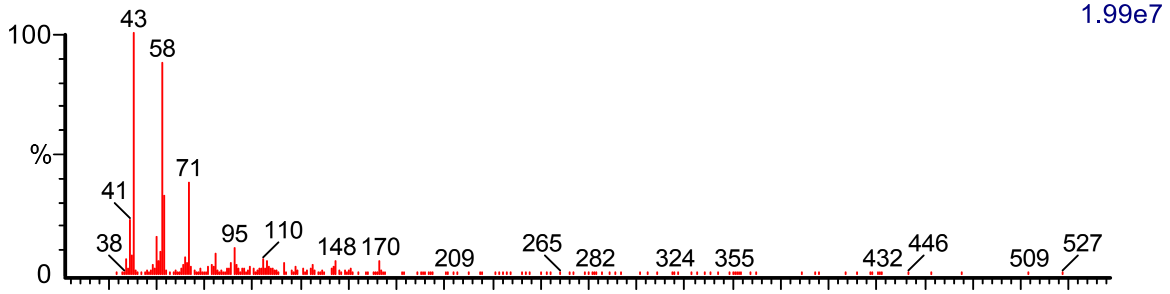

Borneol, acetate (**38**), 8.569 min, (I_r 1293)

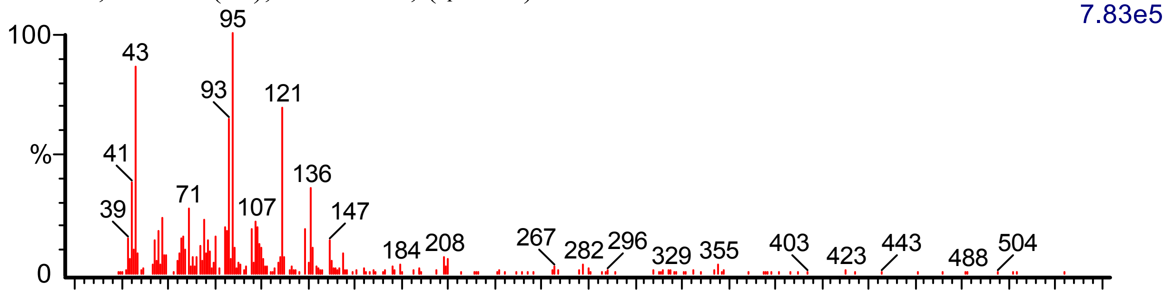

Elemene Isomer (**39**), 8.914 min, (I_r 1343)

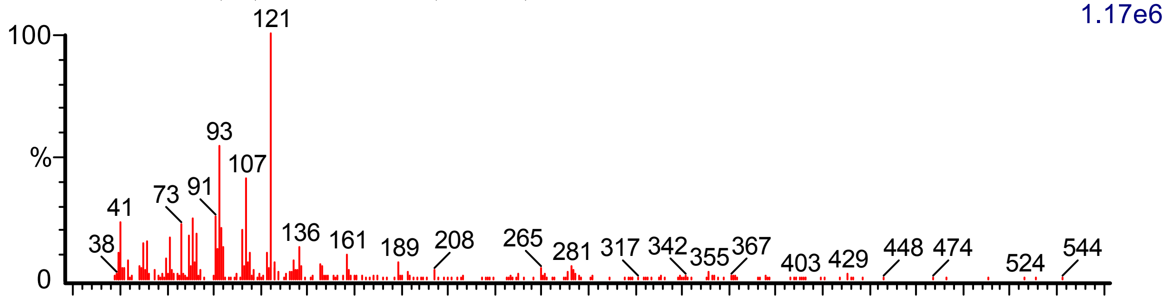

Figure S18. Mass spectra of volatile compounds (continuation)

α-Longipinene (**40**), 9.034 min, (I_r 1361)

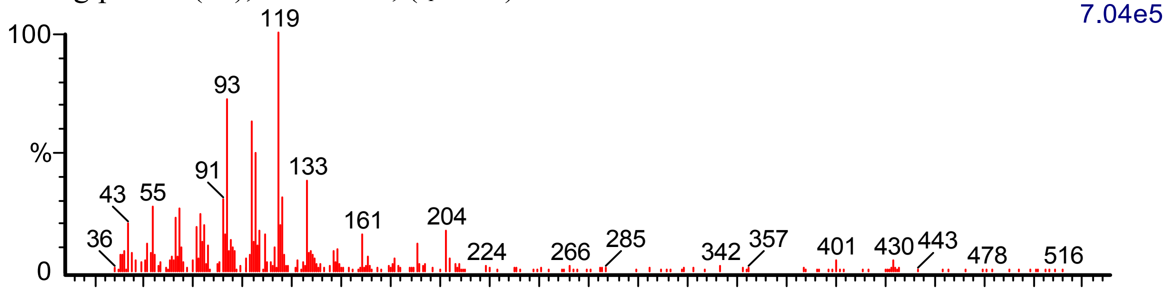

Cyclosativene (**41**), 9.139 min, (I_r 1376)

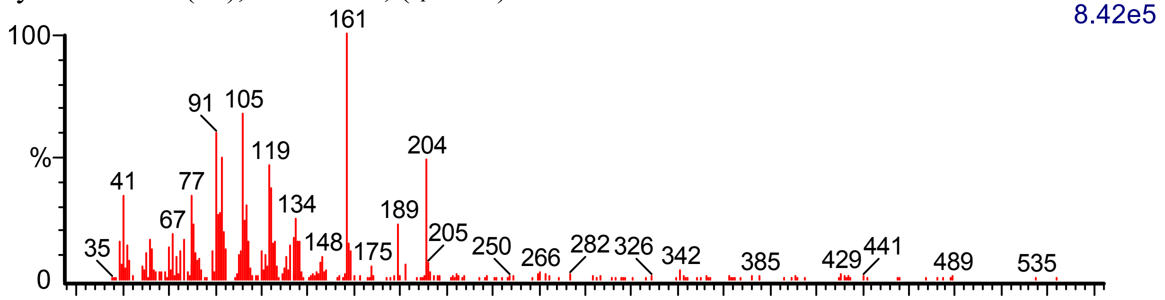

α-Copaene (**42**), 9.192min, (I_r 1374)

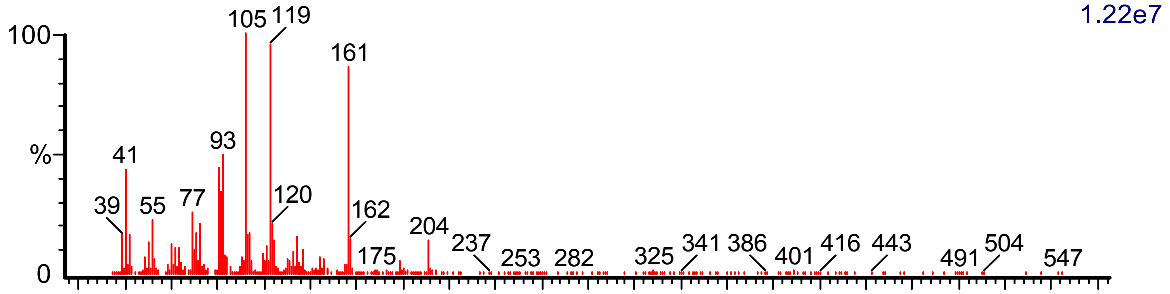

β-Bourbonene (**43**), 9.259 min, (I_r 1385)

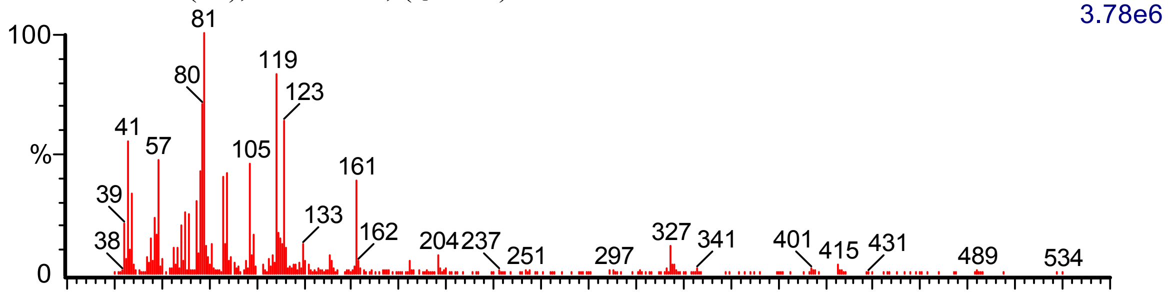

Figure S18. Mass spectra of volatile compounds (continuation)

Sesquithujene (**44**), 9.244 min, (I_r 1392)

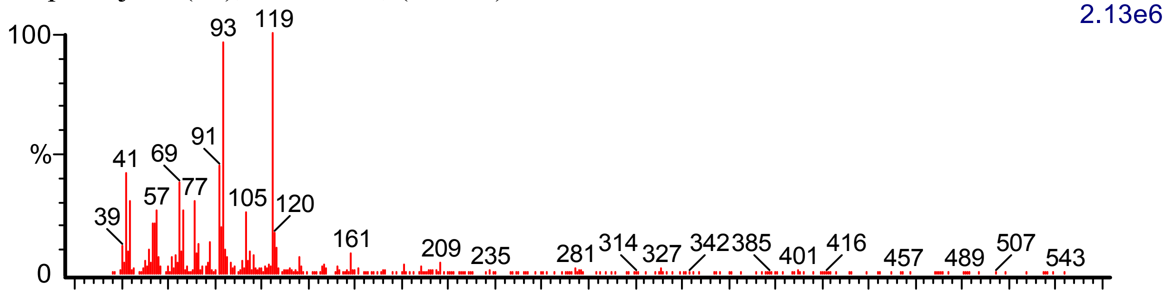

Eugenol methyl ether (**44**), 9.319min, (I_r 1394)

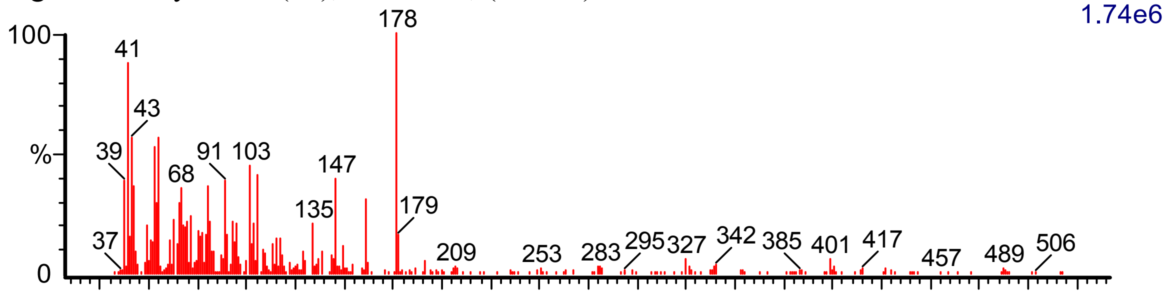

Dodecanal (**46**), 9.327 min, (I_r 404)

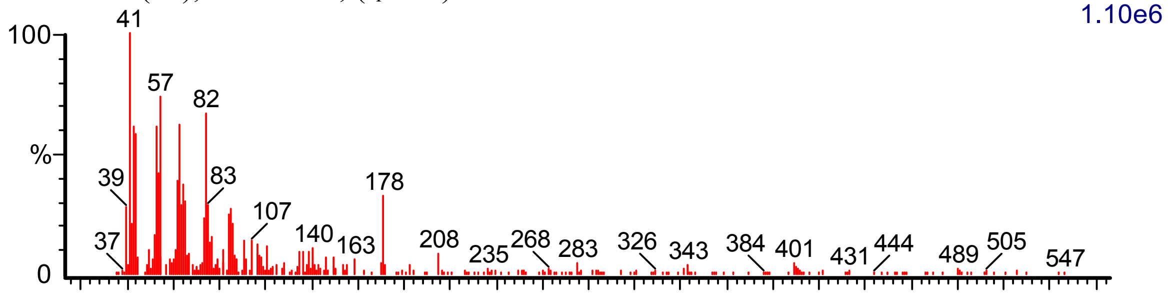

α-Cedrene (**47**), 9.477 min, (I_r 9.469)

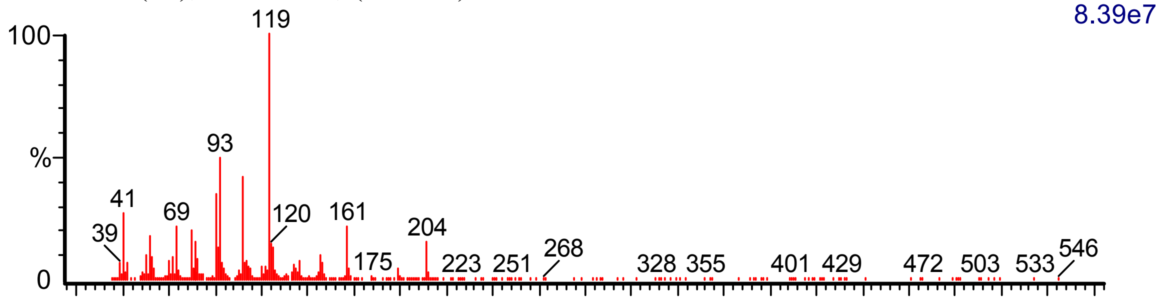

Figure S18. Mass spectra of volatile compounds (continuation)

β-Cedrene (**48**), 9.529 min, (I_r x1426)

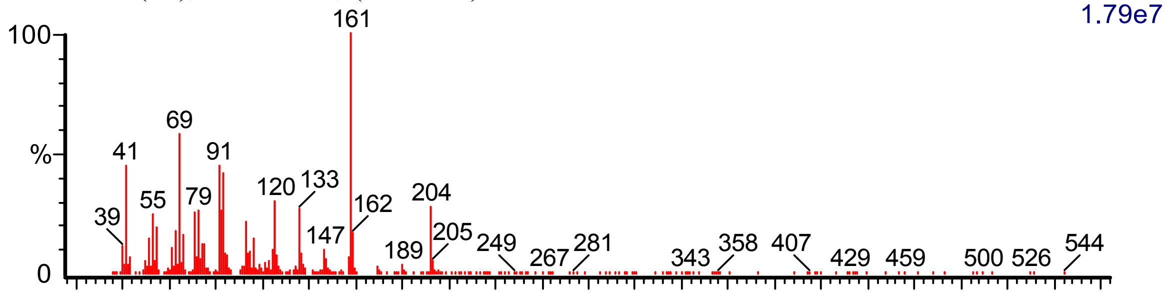

β-Caryophyllene (**49**), 9.499 min, (I_r 1431)

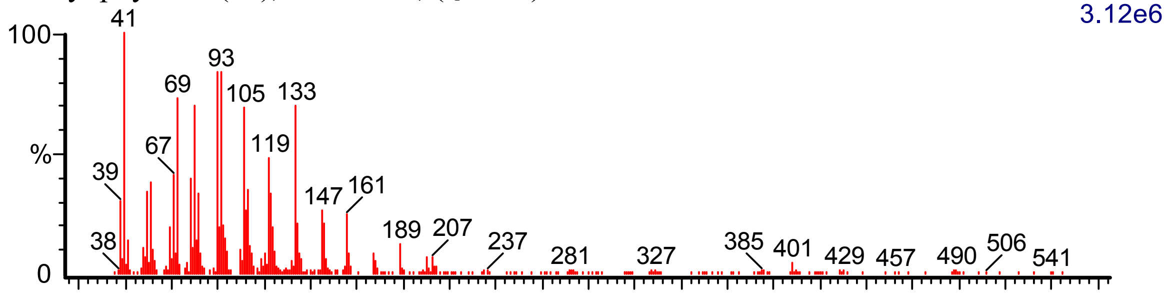

D-Germacrene (**50**), 9.552 min, (I_r 1440)

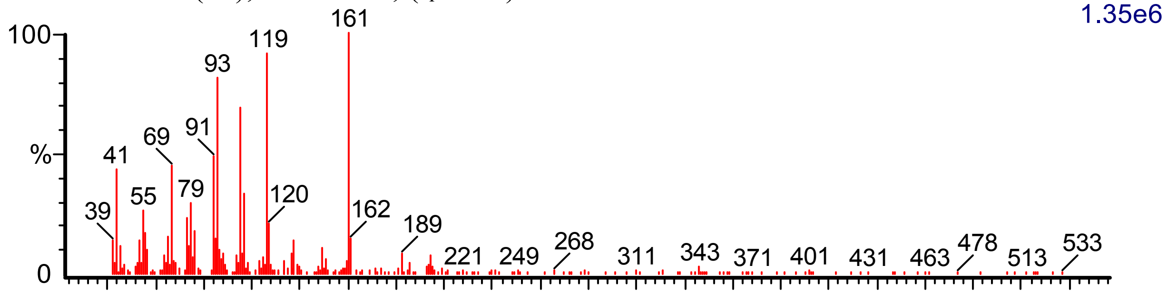

Geranyl acetone (**51**), 9.619 min, (I_r 1450)

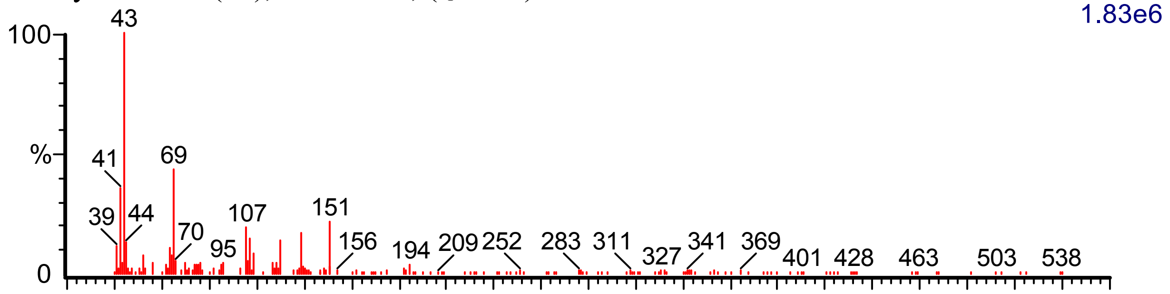

Figure S18. Mass spectra of volatile compounds (continuation)

Prezizaene (**52**), 9.694 min, (I_r 1453)

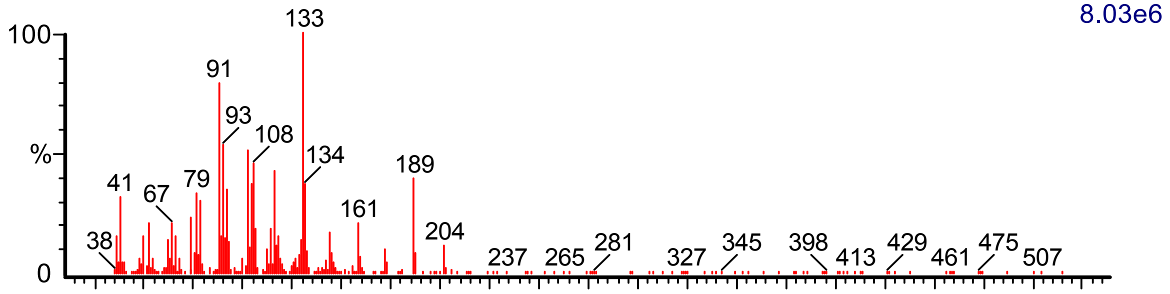

Eremophilene (**53**), 9.740 min, (I_r 1460)

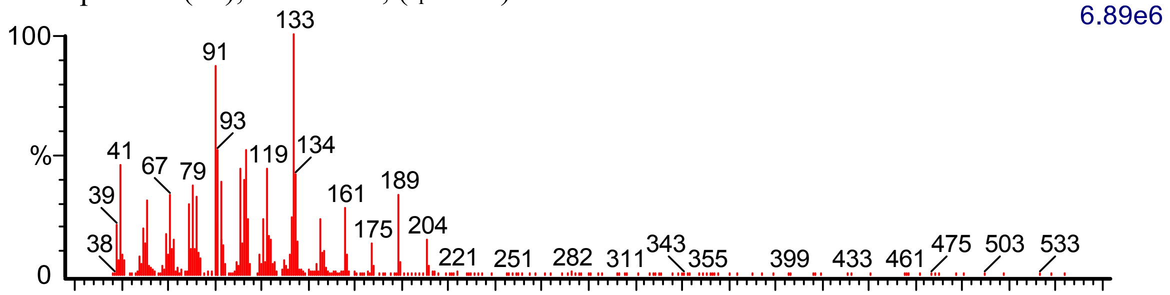

(+)-Valencene (**54**), 9.732 min, (I_r 1468)

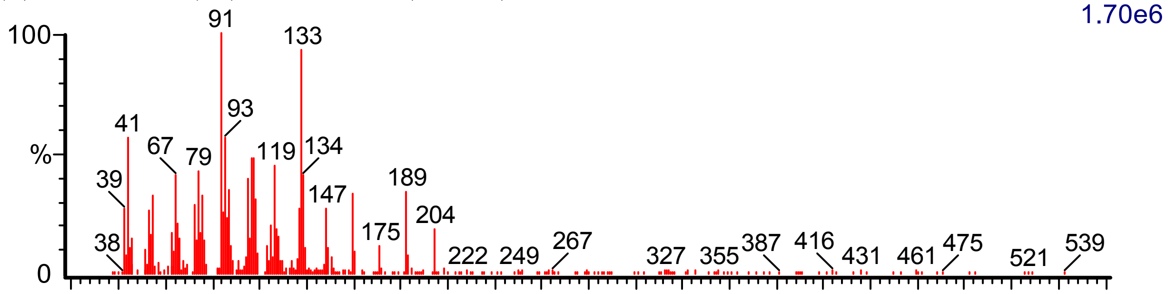

Methyl undecyl ketone (**56**), 9.927 min, (I_r 1482)

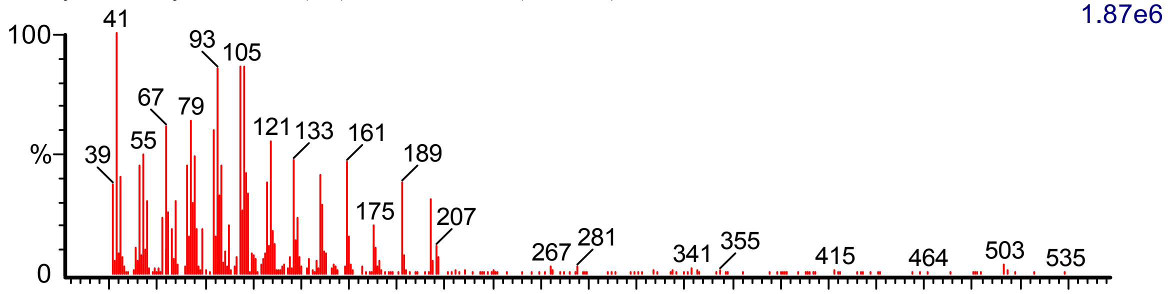

Figure S18. Mass spectra of volatile compounds (continuation)

β-Selinene (**57**), 9.927 min, (I_r 1489)

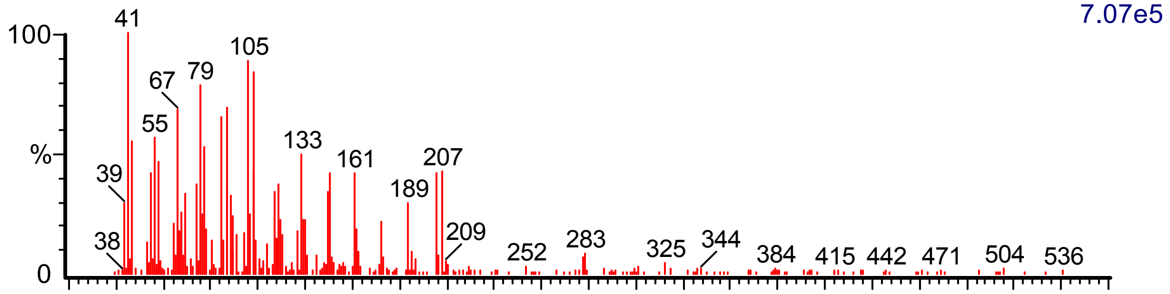

Eremophilene (**58**), 9.935 min, (I_r 1490)

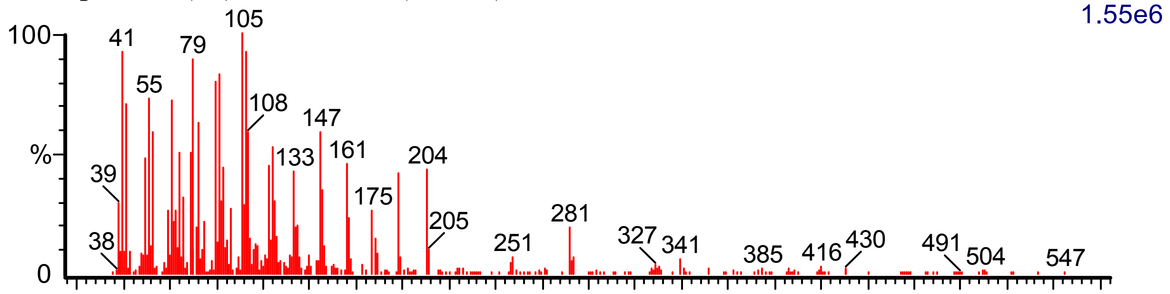

α-Muurolene (**59**), 9.979 min, (I_r 1497)

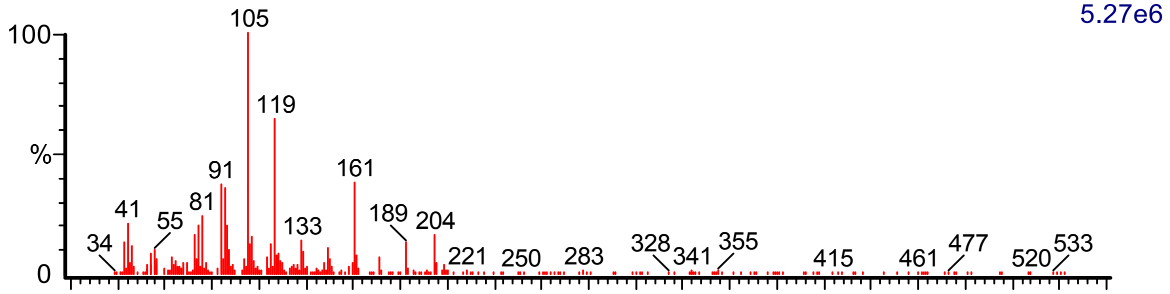

Butylhydroxitoluene (**60**), 10.040 min, (I_r 1507)

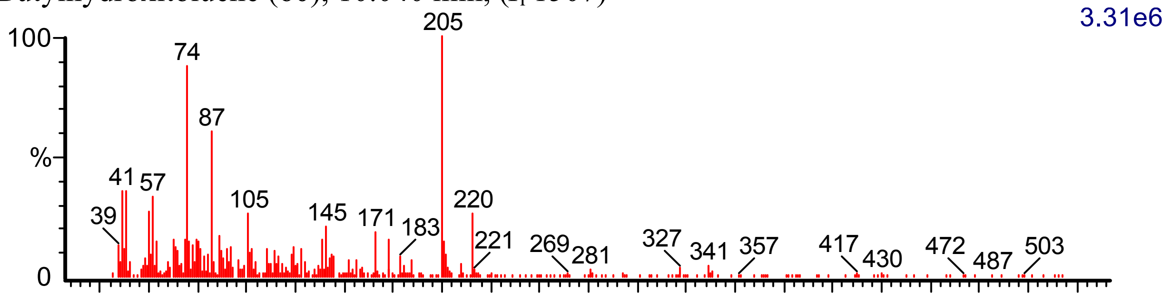

Figure S18. Mass spectra of volatile compounds (continuation)

Dodecanoic acid (**61**), 10.265 min, (*I*$_r$ 1545)

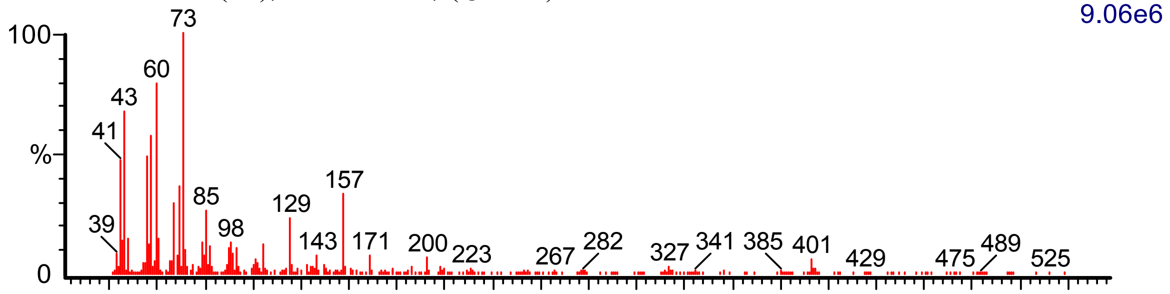

(+)-Nerolidol (**62**), 10.302 min, (*I*$_r$ 1552)

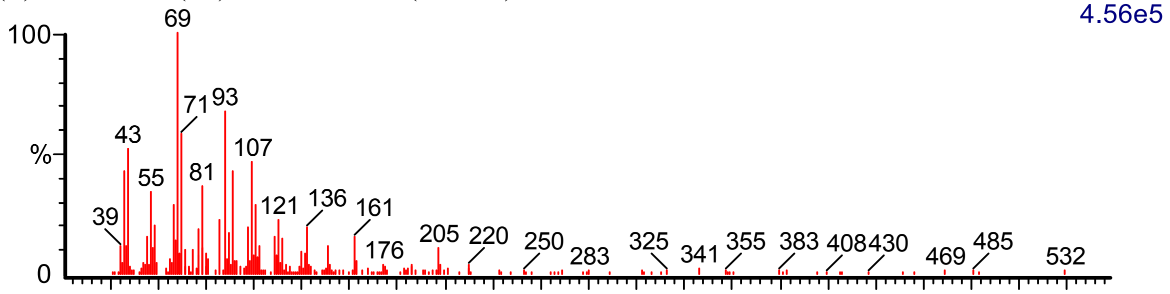

α-Cedrol (**65**), 10.655 min, (*I*$_r$ 1613)

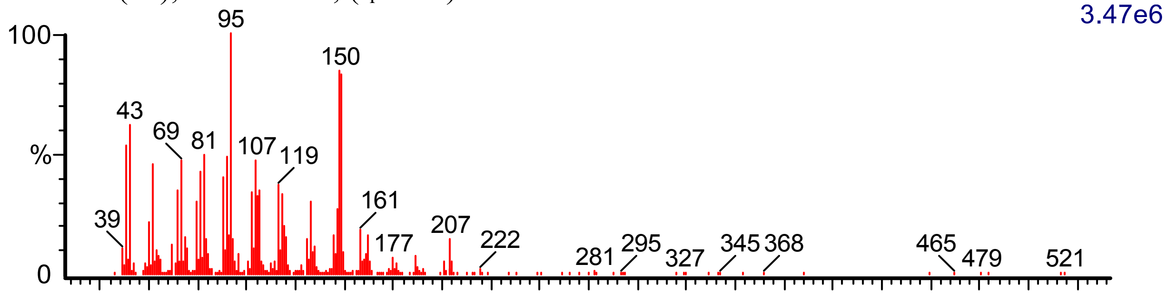

Epicedrol (**66**), 10.655 min, (*I*$_r$ 1613)

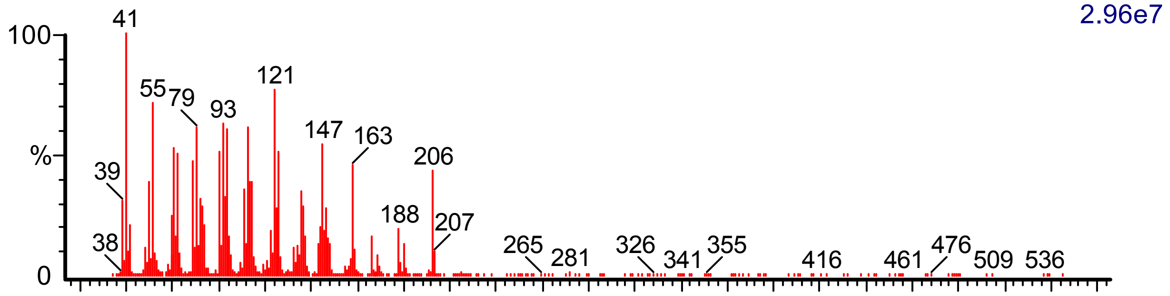

Figure S18. Mass spectra of volatile compounds (continuation)

Cedrol Isomer (**68**), 10.737 min, (I_r 1626)

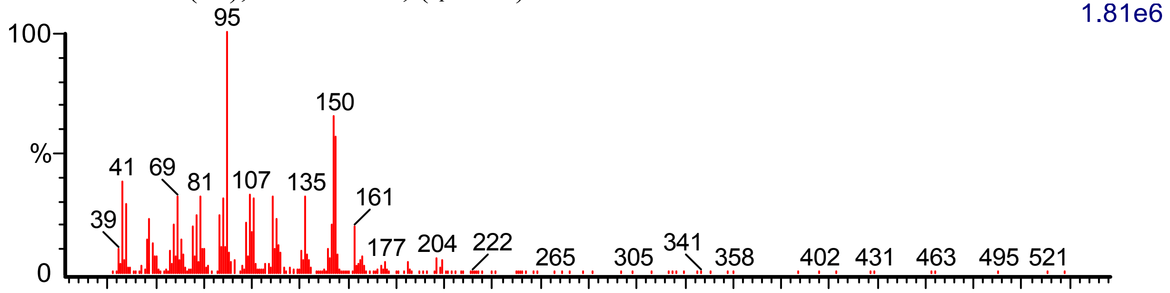

Prezizaan-7-ol (**70**), 10.940 min, (I_r 1662)

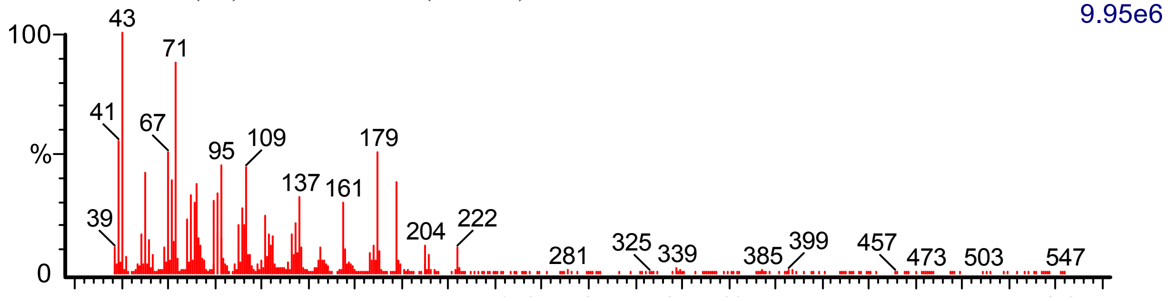

Hexyl salicylate (**71**), 11.000 min, (I_r 1683)

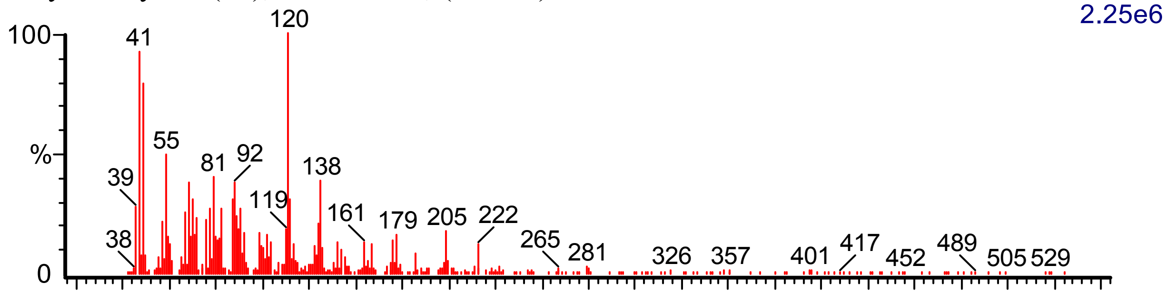

Methyl myristoleate (**72**), 11.127 min, (I_r 1696)

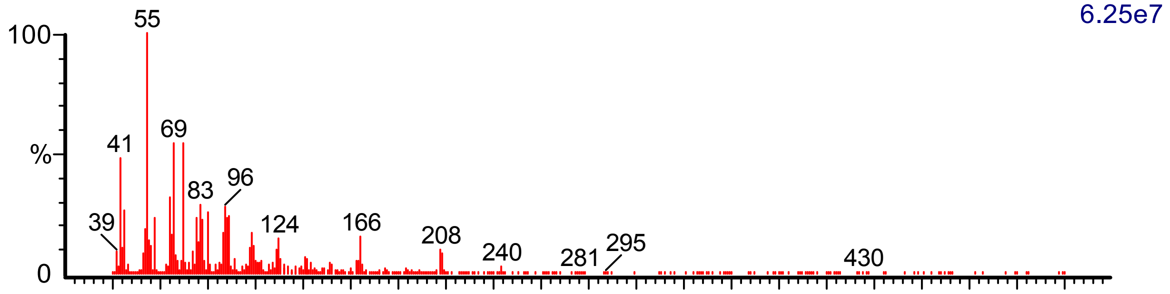

Figure S18. Mass spectra of volatile compounds (continuation)

Methyl myristate (**73**), 11.18min, (I_r 1705)

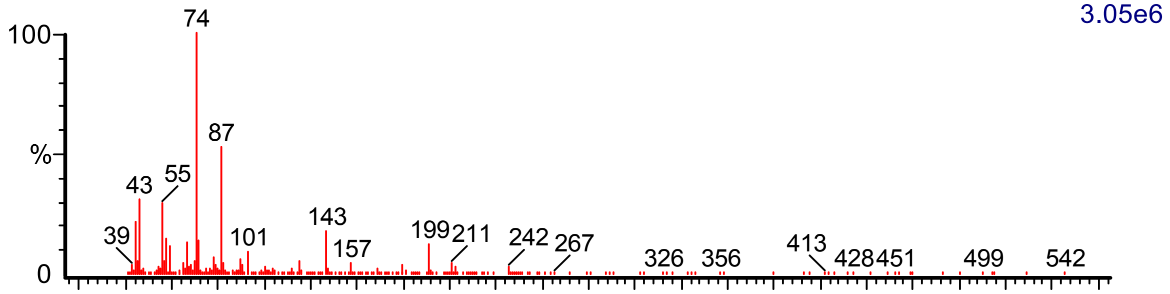

Methyl tetradecanoate (**74**), 11.195 min, (I_r 1708)

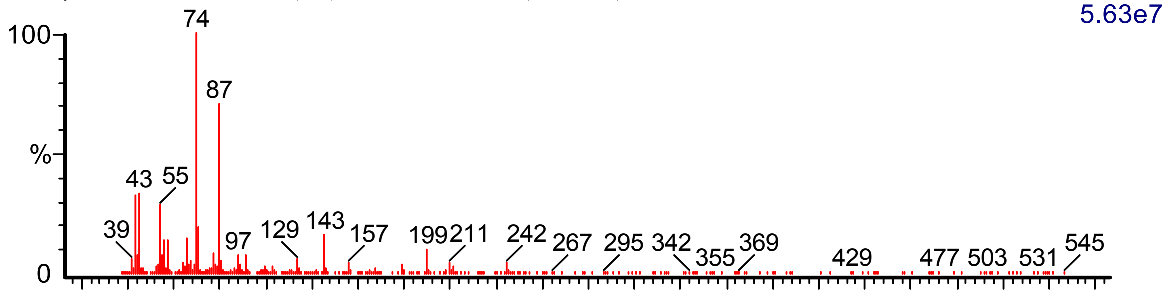

Myristic acid (**75**), 11.487 min, (I_r 1740)

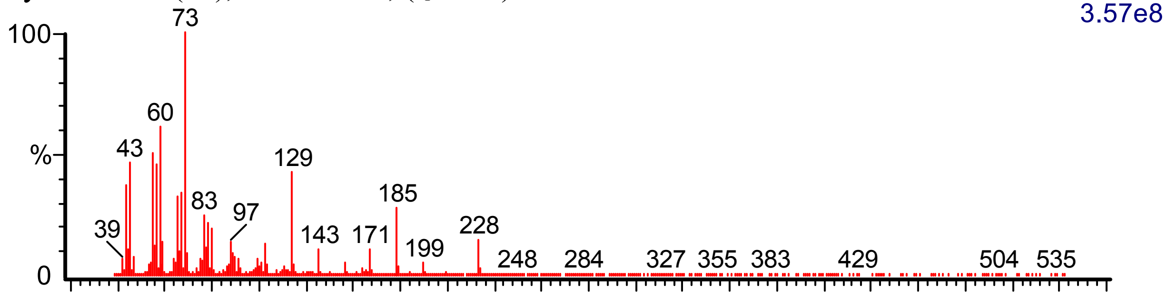

Figure S18. Mass spectra of volatile compounds (continuation)

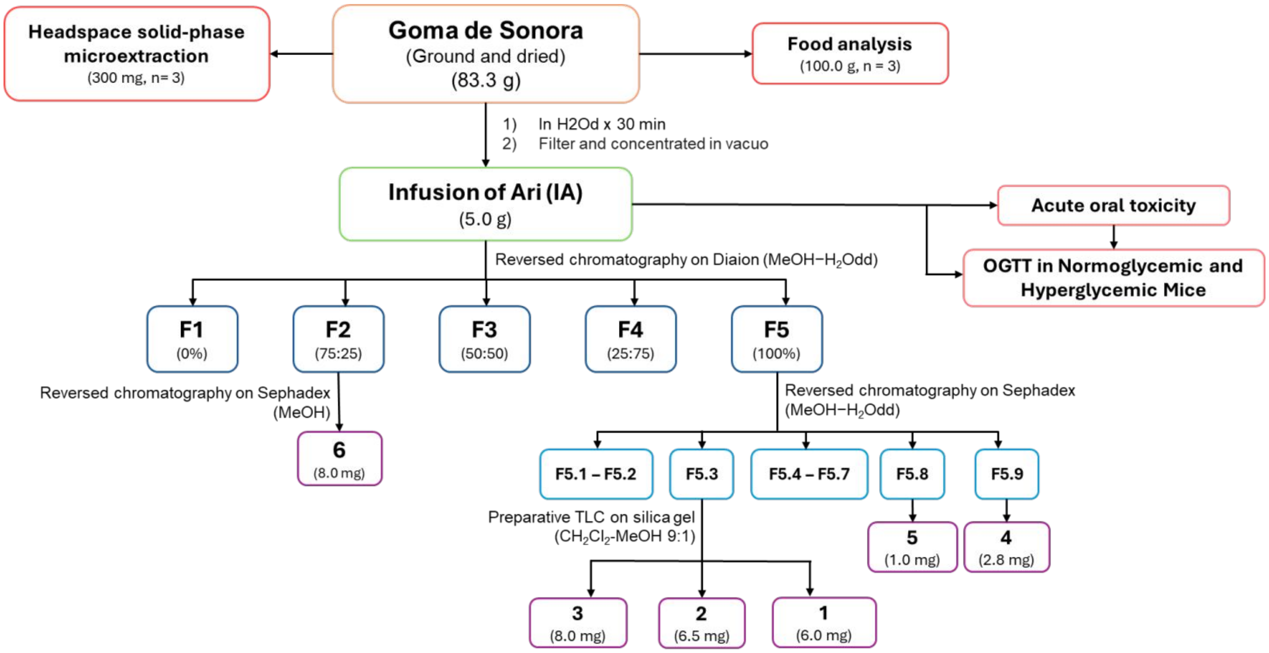

Figure S19. Process of extraction and pharmacological analysis of the arí**.**

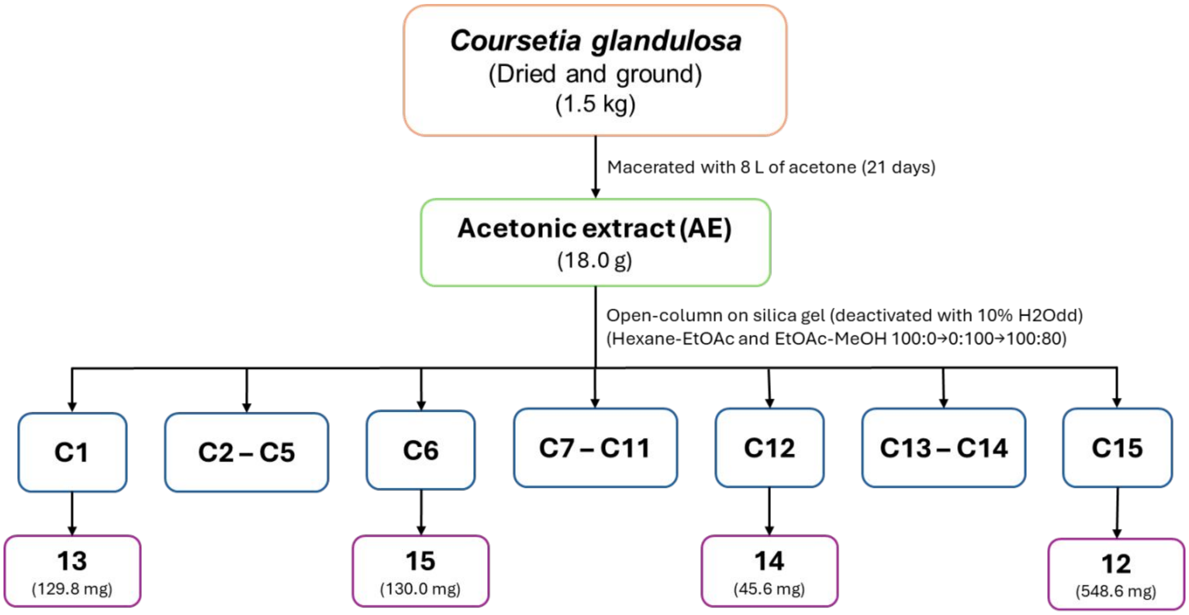

Figure S20. Process of extraction of *C. glandulosa*.

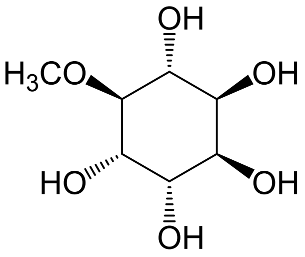

12

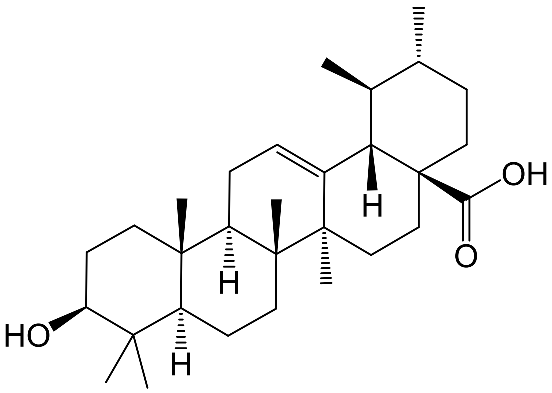

15

Figure S21. Structures of 12 and 15 isolated from *C. glandulosa.*

Table S1. Identified volatile compounds from Sonora gum by HS-SPME/GC-MS

No.	Compound	I_R	PDMS	DVB/CAR/PDMS	PDMS/DVB	CAR/PDMS
				%		
			red	grey	blue	black
16	Methyl amyl ketone	890	-	-	-	0.429
17	Heptanal	900	-	0.872	0.369	0.765
18	No identified	911	-	-	1.587	-
19	Methyl caproate	924	-	-	0.492	0.432
20	2-Methyl-1-octene-3-yne	978	-	2.441	1.028	1.020
21	Sulcatone	985	-	2.231	1.207	1.461
22	Octanal	997	-	0.893	-	0.479
23	Lavender lactone	1038	-	4.527	2.188	1.974
24	Arbusculone	1048	-	0.790	0.504	0.636
25	Methyl heptyl ketone	1084	-		0.855	0.768
26	(2)-Nonanol	1092	-	1.418	0.575	0.754
27	Nonanal	1095	-	2.494	1.409	1.628
28	Limona ketone	1127	-	0.418	0.344	-
29	*p*-Acetotoluene	1182	-	2.095	1.432	1.130
30	No identified	1195	-	3.408	2.550	2.450
31	Lilac Alcohol Isomer	1205	-	0.470	-	-
32	Lilac Alcohol Isomer	1209	-	0.948	-	-
33	(+)-Nordavanone	1224	-	0.568	0.314	0.437
34	Geraniol	1245	-	-	0.239	0.205
35	(*E*)-2-Decenal	1254	-	-	0.269	
36	α-Ionene	1261	-	-	0.336	0.383
37	Methyl nonyl ketone	1289	0.116	-	0.815	0.659
38	Borneol, acetate	1293	0.125	-	-	-

39	Elemene Isomer	1343	0.098	-	-	-
40	α-Longipinene	1361	0.144	-	0.282	0.409
41	Cyclosativene	1376	0.135	-	0.339	0.446
42	α-Copaene	1374	0.628	1.999	0.774	1.151
43	β-Bourbonene	1385	-		-	0.463
44	Sesquithujene	1392	0.218		-	-
45	Eugenol methyl ether	1394	-	0.254	-	0.249
46	Dodecanal	1404	0.206	-	-	-
47	α-Cedrene	1416	-	5.079	4.350	6.056
48	β-Cedrene	1426	1.191	0.656	0.579	1.401
49	β-Caryophyllene	1431	0.654	-	-	-
50	D-Germacrene	1440	0.214	-	-	-
51	Geranyl acetone	1450	0.105	-	-	-
52	Prezizaene	1453	-	-	0.569	0.828
53	Eremophilene	1460	-	-	0.582	-
54	(+)-Valencene	1468	0.323	-	-	-
55	No identified	1470	-	-	1.042	-
56	Methyl undecyl keton	1482	-	-	1.219	0.295
57	β-Selinene	1489	0.194	-	-	-
58	Eremophilene	1490	-	-	0.270	-
59	α-Muurolene	1497	0.441	-	0.172	0.428
60	Butylhydroxitoluene	1507	-	-	0.506	-
61	Dodecanoic acid	1545	-	-	0.607	-

62	(+)-Nerolidol	1552	-	1.598	0.779	1.085
63	No identified	1609	-	14.032	-	9.827
64	No identified	1612	-	0.000	5.464	-
65	α-Cedrol	1613	-	18.064	6.025	9.243
66	No identified	1621	6.457	-	-	-
67	Epicedrol	1623	8.902	-	-	-
68	Cedrol Isomer	1626	3.703	6.761	2.008	2.210
69	No identified	1661	-	-	-	1.590
70	Prezizaan-7-ol	1662	-	-	0.961	-
71	Hexyl salicylate	1683	0.422	-	-	-
72	Methyl myristoleate	1696	7.328	-	-	3.360
73	Methyl myristate	1705	3.691	4.499	3.776	-
74	Methyl tetradecanoate	1708	-	-	1.960	1.870
75	Myristic acid	1740	62.203	12.897	45.882	40.663
			**97.50**	**89.41**	**94.66**	**96.76**

Table S2. Lorke assay data

**IA**			
**Fase I**		**Fase II**	
Dose (mg/kg)	Mortality	Dose (mg/kg)	Mortality
10	0/3	1600	0/3
100	0/3	2900	0/3
1000	0/3	5000	0/3
